# Cognitive correlates of antisaccade behaviour across multiple neurodegenerative diseases

**DOI:** 10.1093/braincomms/fcad049

**Published:** 2023-03-02

**Authors:** Heidi C Riek, Donald C Brien, Brian C Coe, Jeff Huang, Julia E Perkins, Rachel Yep, Paula M McLaughlin, Joseph B Orange, Alicia J Peltsch, Angela C Roberts, Malcolm A Binns, Wendy Lou, Agessandro Abrahao, Stephen R Arnott, Derek Beaton, Sandra E Black, Dar Dowlatshahi, Elizabeth Finger, Corinne E Fischer, Andrew R Frank, David A Grimes, Sanjeev Kumar, Anthony E Lang, Jane M Lawrence-Dewar, Jennifer L Mandzia, Connie Marras, Mario Masellis, Stephen H Pasternak, Bruce G Pollock, Tarek K Rajji, Demetrios J Sahlas, Gustavo Saposnik, Dallas P Seitz, Christen Shoesmith, Thomas D L Steeves, Stephen C Strother, Kelly M Sunderland, Richard H Swartz, Brian Tan, David F Tang-Wai, Maria Carmela Tartaglia, John Turnbull, Lorne Zinman, Douglas P Munoz, the ONDRI Investigators, Sabrina Adamo, Rob Bartha, Courtney Berezuk, Alanna Black, Michael Borrie, Susan Bronskill, Dennis Bulman, Leanne Casaubon, Ben Cornish, Sherif Defrawy, Allison Dilliott, Roger A Dixon, Sali Farhan, Frederico Faria, Julia Fraser, Morris Freedman, Mahdi Ghani, Barry Greenberg, Hassan Haddad, Ayman Hassan, Wendy Hatch, Rob Hegele, Melissa Holmes, Chris Hudson, Mandar Jog, Peter Kleinstiver, Donna Kwan, Elena Leontieva, Brian Levine, Efrem Mandelcorn, Ed Margolin, Bill McIlroy, Manuel Montero-Odasso, David Munoz, Nuwan Nanayakkara, Miracle Ozzoude, Joel Ramirez, Natalie Rashkovan, John Robinson, Ekaterina Rogaeva, Yanina Sarquis Adamson, Christopher Scott, Michael Strong, Sujeevini Sujanthan, Sean Symons, Athena Theyers, Angela Troyer, Karen Van Ooteghem, John Woulfe, Mojdeh Zamyadi

**Affiliations:** Centre for Neuroscience Studies, Queen’s University, Kingston, Ontario K7L 3N6Canada; Centre for Neuroscience Studies, Queen’s University, Kingston, Ontario K7L 3N6Canada; Centre for Neuroscience Studies, Queen’s University, Kingston, Ontario K7L 3N6Canada; Centre for Neuroscience Studies, Queen’s University, Kingston, Ontario K7L 3N6Canada; Centre for Neuroscience Studies, Queen’s University, Kingston, Ontario K7L 3N6Canada; Centre for Neuroscience Studies, Queen’s University, Kingston, Ontario K7L 3N6Canada; Nova Scotia Health, Halifax, Nova Scotia B3S 0H6, Canada; Department of Medicine (Geriatrics), Dalhousie University, Halifax, Nova Scotia B3H 2Y9, Canada; Department of Psychology and Neuroscience, Dalhousie University, Halifax, Nova Scotia B3H 4R2, Canada; School of Communication Sciences and Disorders, Faculty of Health Sciences, Western University, London, Ontario N6G 1H1, Canada; Canadian Centre for Activity and Aging, Faculty of Health Sciences, Western University, London, Ontario N6G 1H1, Canada; Faculty of Engineering and Applied Science, Queen’s University, Kingston Ontario K7L 3N6, Canada; School of Communication Sciences and Disorders, Faculty of Health Sciences, Western University, London, Ontario N6G 1H1, Canada; Department of Computer Science, Western University, London, Ontario N6A 5B7, Canada; Rotman Research Institute, Baycrest Centre, North York, Ontario M6A 2E1, Canada; Dalla Lana School of Public Health, University of Toronto, Toronto, Ontario M5T 3M7, Canada; Dalla Lana School of Public Health, University of Toronto, Toronto, Ontario M5T 3M7, Canada; Division of Neurology, Department of Medicine, Sunnybrook Health Sciences Centre and University of Toronto, Toronto, Ontario M5S 3H2, Canada; Hurvitz Brain Sciences Program, Sunnybrook Research Institute, University of Toronto, Toronto, Ontario M4N 3M5, Canada; Rotman Research Institute, Baycrest Centre, North York, Ontario M6A 2E1, Canada; Rotman Research Institute, Baycrest Centre, North York, Ontario M6A 2E1, Canada; Division of Neurology, Department of Medicine, Sunnybrook Health Sciences Centre and University of Toronto, Toronto, Ontario M5S 3H2, Canada; Hurvitz Brain Sciences Program, Sunnybrook Research Institute, University of Toronto, Toronto, Ontario M4N 3M5, Canada; Department of Medicine (Neurology), University of Ottawa, Ottawa, Ontario K1H 8M5, Canada; Ottawa Hospital Research Institute, Ottawa, Ontario K1Y 4E9, Canada; Department of Clinical Neurological Sciences, Schulich School of Medicine and Dentistry, Western University, London, Ontario N6A 3K7, Canada; Keenan Research Centre for Biomedical Science, St. Michael’s Hospital, Toronto, Ontario M5B 1W8, Canada; Department of Medicine (Neurology), University of Ottawa, Ottawa, Ontario K1H 8M5, Canada; Bruyere Research Institute, Ottawa, Ontario K1R 6M1, Canada; Department of Medicine (Neurology), University of Ottawa, Ottawa, Ontario K1H 8M5, Canada; Ottawa Hospital Research Institute, Ottawa, Ontario K1Y 4E9, Canada; University of Ottawa Brain and Mind Research Institute, University of Ottawa, Ottawa, Ontario K1H 8M5, Canada; Campbell Family Mental Health Research Institute, Centre for Addiction and Mental Health, Toronto, Ontario M6J 1H4, Canada; Department of Psychiatry, Temerty Faculty of Medicine, University of Toronto, Toronto, Ontario M5S 1A8, Canada; Division of Neurology, Department of Medicine, University of Toronto, Toronto, Ontario M5S 3H2, Canada; Edmond J. Safra Program in Parkinson’s Disease, Toronto Western Hospital, Toronto, Ontario M5T 2S8, Canada; Thunder Bay Regional Health Research Institute, Thunder Bay, Ontario P7B 7A5, Canada; Department of Clinical Neurological Sciences, Schulich School of Medicine and Dentistry, Western University, London, Ontario N6A 3K7, Canada; London Health Sciences Centre, London, Ontario N6A 5W9, Canada; Division of Neurology, Department of Medicine, University of Toronto, Toronto, Ontario M5S 3H2, Canada; Edmond J. Safra Program in Parkinson’s Disease, Toronto Western Hospital, Toronto, Ontario M5T 2S8, Canada; Hurvitz Brain Sciences Program, Sunnybrook Research Institute, University of Toronto, Toronto, Ontario M4N 3M5, Canada; Division of Neurology, Department of Medicine, University of Toronto, Toronto, Ontario M5S 3H2, Canada; Cognitive and Movement Disorders Clinic, Sunnybrook Health Sciences Centre, Toronto, Ontario M4N 3M5, Canada; Department of Clinical Neurological Sciences, Schulich School of Medicine and Dentistry, Western University, London, Ontario N6A 3K7, Canada; Robarts Research Institute, Schulich School of Medicine and Dentistry, Western University, London, Ontario N6A 5B7, Canada; Cognitive Neurology and Alzheimer’s Disease Research Centre, Parkwood Institute, St. Joseph’s Health Care, London, Ontario N6A 4V2, Canada; Campbell Family Mental Health Research Institute, Centre for Addiction and Mental Health, Toronto, Ontario M6J 1H4, Canada; Department of Psychiatry, Temerty Faculty of Medicine, University of Toronto, Toronto, Ontario M5S 1A8, Canada; Department of Psychiatry, Temerty Faculty of Medicine, University of Toronto, Toronto, Ontario M5S 1A8, Canada; Toronto Dementia Research Alliance, University of Toronto, Toronto, Ontario M5S 1A8, Canada; Department of Medicine, Faculty of Health Sciences, McMaster University, Hamilton, Ontario L8N 3Z5, Canada; Division of Neurology, Department of Medicine, University of Toronto, Toronto, Ontario M5S 3H2, Canada; Department of Psychiatry, Cumming School of Medicine, University of Calgary, Calgary, Alberta T2N 1N4, Canada; Department of Clinical Neurological Sciences, Schulich School of Medicine and Dentistry, Western University, London, Ontario N6A 3K7, Canada; London Health Sciences Centre, London, Ontario N6A 5W9, Canada; Division of Neurology, Department of Medicine, University of Toronto, Toronto, Ontario M5S 3H2, Canada; Division of Neurology, St. Michael’s Hospital, Toronto, Ontario M5B 1W8, Canada; Rotman Research Institute, Baycrest Centre, North York, Ontario M6A 2E1, Canada; Department of Medical Biophysics, University of Toronto, Toronto, Ontario M5G 1L7, Canada; Rotman Research Institute, Baycrest Centre, North York, Ontario M6A 2E1, Canada; Division of Neurology, Department of Medicine, Sunnybrook Health Sciences Centre and University of Toronto, Toronto, Ontario M5S 3H2, Canada; Hurvitz Brain Sciences Program, Sunnybrook Research Institute, University of Toronto, Toronto, Ontario M4N 3M5, Canada; Rotman Research Institute, Baycrest Centre, North York, Ontario M6A 2E1, Canada; Division of Neurology, Department of Medicine, University of Toronto, Toronto, Ontario M5S 3H2, Canada; University Health Network Memory Clinic, Krembil Brain Institute, Toronto Western Hospital, Toronto, Ontario M5T 2S8, Canada; University Health Network Memory Clinic, Krembil Brain Institute, Toronto Western Hospital, Toronto, Ontario M5T 2S8, Canada; Tanz Centre for Research in Neurodegenerative Diseases, University of Toronto, Toronto, Ontario M5S 1A8, Canada; Department of Medicine, Faculty of Health Sciences, McMaster University, Hamilton, Ontario L8N 3Z5, Canada; Division of Neurology, Department of Medicine, Sunnybrook Health Sciences Centre and University of Toronto, Toronto, Ontario M5S 3H2, Canada; Hurvitz Brain Sciences Program, Sunnybrook Research Institute, University of Toronto, Toronto, Ontario M4N 3M5, Canada; Centre for Neuroscience Studies, Queen’s University, Kingston, Ontario K7L 3N6Canada; Department of Biomedical and Molecular Sciences, Queen’s University, Kingston, Ontario K7L 3N6, Canada

**Keywords:** antisaccade, prosaccade, cognitive impairment, neurodegenerative disease, dementia

## Abstract

Oculomotor tasks generate a potential wealth of behavioural biomarkers for neurodegenerative diseases. Overlap between oculomotor and disease-impaired circuitry reveals the location and severity of disease processes via saccade parameters measured from eye movement tasks such as prosaccade and antisaccade. Existing studies typically examine few saccade parameters in single diseases, using multiple separate neuropsychological test scores to relate oculomotor behaviour to cognition; however, this approach produces inconsistent, ungeneralizable results and fails to consider the cognitive heterogeneity of these diseases. Comprehensive cognitive assessment and direct inter-disease comparison are crucial to accurately reveal potential saccade biomarkers. We remediate these issues by characterizing 12 behavioural parameters, selected to robustly describe saccade behaviour, derived from an interleaved prosaccade and antisaccade task in a large cross-sectional data set comprising five disease cohorts (Alzheimer’s disease/mild cognitive impairment, amyotrophic lateral sclerosis, frontotemporal dementia, Parkinson’s disease, and cerebrovascular disease; *n* = 391, age 40–87) and healthy controls (*n* = 149, age 42–87). These participants additionally completed an extensive neuropsychological test battery. We further subdivided each cohort by diagnostic subgroup (for Alzheimer’s disease/mild cognitive impairment and frontotemporal dementia) or degree of cognitive impairment based on neuropsychological testing (all other cohorts). We sought to understand links between oculomotor parameters, their relationships to robust cognitive measures, and their alterations in disease. We performed a factor analysis evaluating interrelationships among the 12 oculomotor parameters and examined correlations of the four resultant factors to five neuropsychology-based cognitive domain scores. We then compared behaviour between the abovementioned disease subgroups and controls at the individual parameter level. We theorized that each underlying factor measured the integrity of a distinct task-relevant brain process. Notably, Factor 3 (voluntary saccade generation) and Factor 1 (task disengagements) significantly correlated with attention/working memory and executive function scores. Factor 3 also correlated with memory and visuospatial function scores. Factor 2 (pre-emptive global inhibition) correlated only with attention/working memory scores, and Factor 4 (saccade metrics) correlated with no cognitive domain scores. Impairment on several mostly antisaccade-related individual parameters scaled with cognitive impairment across disease cohorts, while few subgroups differed from controls on prosaccade parameters. The interleaved prosaccade and antisaccade task detects cognitive impairment, and subsets of parameters likely index disparate underlying processes related to different cognitive domains. This suggests that the task represents a sensitive paradigm that can simultaneously evaluate a variety of clinically relevant cognitive constructs in neurodegenerative and cerebrovascular diseases and could be developed into a screening tool applicable to multiple diagnoses.

## Teaser

Riek *et al*. report that oculomotor parameters derived from prosaccade/antisaccade assessment in a large cohort (*n* = 391) of neurodegenerative disease patients load on four factors corresponding to distinct brain processes and correlate differentially to cognitive domains. Additionally, cognitively impaired patients were differentiated from cognitively normal patients and controls on individual parameters.

## Graphical Abstract

**Graphical Abstract:**
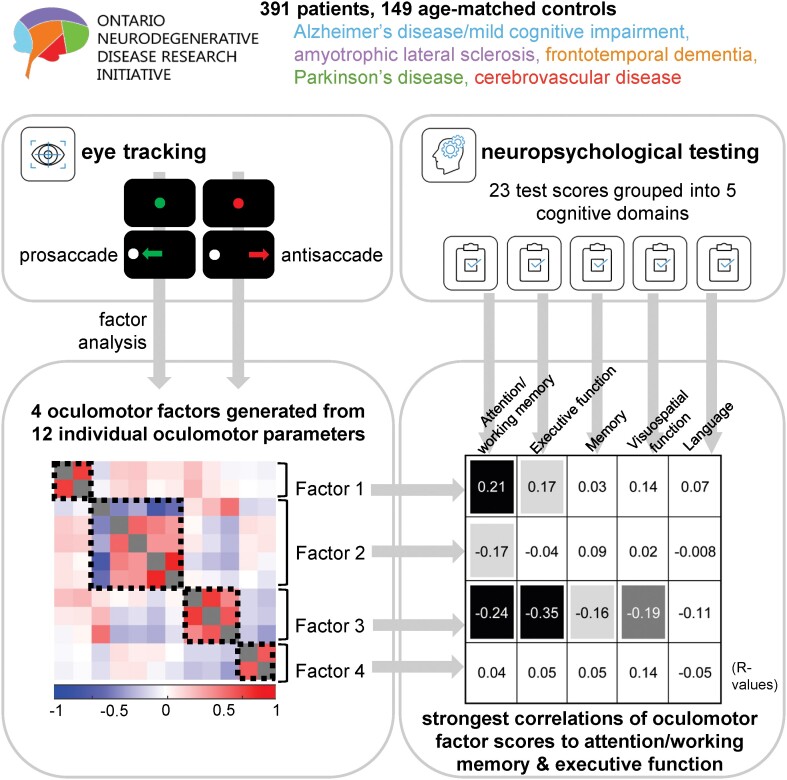

## Introduction

Neurodegenerative and cerebrovascular diseases display significant clinical and pathological variability, making them challenging to differentiate and treat. This difficulty is partly due to the complexity of predominantly sporadic diseases that either present with or eventually involve cognitive impairment. Although Alzheimer’s disease, mild cognitive impairment (MCI), and behavioural variant frontotemporal dementia (bvFTD) always involve cognitive impairment, it can be heterogeneous in degree and domain, and there may be clinical and/or pathological overlap between subgroups.^1,2^ Furthermore, movement disorders such as Parkinson’s disease, progressive supranuclear palsy (PSP), and amyotrophic lateral sclerosis can engender variable cognitive impairment in some individuals, while the presence of motor symptoms additionally complicates differential diagnosis.^3–5^ Finally, cerebrovascular disease can affect cognitive capabilities and may interact with various neurodegenerative pathologies (e.g. Alzheimer’s disease) as they progress.^6^ This spectrum of cognitive impairment between and within neurodegenerative and cerebrovascular diseases may result from a spectrum of underlying pathologies or from concomitant pathologies.^2,7–9^ Ultimately, these issues obstruct accurate diagnosis and appropriate, timely treatment of neurodegenerative diseases. Therefore, a cost-effective, rapid screening tool to simultaneously characterize both motor and cognitive dysfunction would prove highly advantageous to resolving them.

Assessment of oculomotor behaviour generally, and saccadic eye movements especially, has long been used to evaluate neurological dysfunction due to extensive overlap between saccade circuitry and the diffuse network of brain regions affected by neurological diseases.^10^ More recently, oculomotor tasks have been proposed as a source of biomarkers for various neurological conditions, including neurodegenerative diseases, which have frequently been studied in context of oculomotor deficiencies.^11^ Notably, oculomotor tasks can accurately gauge the integrity of both motor and cognitive circuits, making them ideal paradigms to describe and differentiate the complex symptomology of neurodegenerative disease. However, existing studies often encompass only a single disease (e.g. Parkinson’s disease) or closely related diseases (e.g. Alzheimer’s disease and MCI) and involve relatively few participants in narrow ranges of disease stage.^12,13^ This approach provides limited information about the heterogeneity within and similarity between diseases. It is therefore crucial to describe oculomotor behaviour across the spectrum of neurodegenerative and cerebrovascular disease on a larger scale.

The antisaccade task^14^ is a widespread task used to evaluate cognitive control in which participants must suppress the automatic response to look at a peripherally appearing visual stimulus and instead generate a voluntary saccade in the opposite direction. Successful performance requires the recruitment of higher-order cognitive circuits comprising parts of the frontal and parietal cortices and basal ganglia^15–19^ and is usually considered a hallmark of intact executive function. In contrast, the prosaccade task requires that the participant look at the peripheral stimulus, a very automated behaviour that assesses the integrity of the visual and motor components of the circuitry. Numerous studies have examined prosaccade and antisaccade behaviour in neurodegeneration but often report only a few outcome parameters such as reaction times and error rates. Although error rates are a well-established correlate of neuropsychological tests of executive function^20,21^ and provide important insight into the function of frontostriatal circuitry, a variety of other potentially valuable behavioural measures can be extracted from these tasks, and their relationships to one another and to aspects of cognitive processing are less understood. Furthermore, while correlations of antisaccade error rates and reaction times to neuropsychological tests are frequent, studies commonly evaluate many individual neuropsychological test scores and may therefore produce inconsistent associations within cognitive domains (e.g. correlations to some tests of executive function, but not others).

To address the limitations of existing studies of prosaccade and antisaccade behaviour in neurodegenerative disease, link antisaccade performance to standardized neuropsychological test scores, and more fully characterize saccade behaviour in neurodegeneration, we analysed 12 parameters measured from an interleaved prosaccade and antisaccade task (IPAST). This task included both prosaccade and antisaccade trials presented in pseudo-random order. For all analyses, we used a sample of neurodegenerative and cerebrovascular disease participants collected by the Ontario Neurodegenerative Disease Research Initiative (ONDRI),^22,23^ a multi-site, longitudinal, observational study involving five different disease cohorts: (i) Alzheimer’s disease and amnestic MCI; (ii) amyotrophic lateral sclerosis; (iii) frontotemporal dementia (FTD); (iv) Parkinson’s disease; and (v) cerebrovascular disease. Participants underwent a variety of meticulous assessments, including clinical, neuropsychological^24^ and eye-tracking tests. The ONDRI protocol thus affords a unique opportunity to examine how saccade behaviour is altered between and within diseases using robust measures of cognitive impairment and domain-specific processing derived from multiple neuropsychological test scores. First, we perform exploratory factor analysis, which generates a data-driven factor structure, to evaluate the constructs underlying oculomotor behaviour in the prosaccade and antisaccade tasks. We then correlate the resultant oculomotor factor scores to five different cognitive domains: attention/working memory, executive function, language, memory, and visuospatial function, which were measured using consolidated neuropsychological test results. Finally, we compare ONDRI participants’ behaviour on several discrete parameters to data from healthy age-matched control participants who were collected outside ONDRI but completed the same IPAST task.^25^

This study therefore possesses several advantages over previous work describing antisaccade behaviour in neurodegeneration, including (i) insight into the associations between commonly and rarely reported antisaccade outcome parameters, potentially illuminating their shared and discrete circuitry; (ii) enhancing links between prosaccade and antisaccade behaviour and specific aspects of cognitive processing via robust neuropsychology measures encompassing multiple test scores; and (iii) using a large cohort of multiple neurodegenerative and cerebrovascular diseases and varying levels of cognitive impairment, enabling direct examination and comparison of both. The goal of the current study is to understand how IPAST behaviour may be affected by diagnosis and cognitive impairment, whether underlying factors may explain relationships between IPAST parameters and relate to the function of distinct brain circuits, and how these underlying factors relate to dissociable aspects of cognitive processing.

## Materials and methods

### Participants

This study was approved by the Queen’s University Health Sciences and Affiliated Teaching Hospitals Research Ethics Board and research ethics committees at all participating ONDRI recruitment sites.^23^ The study is in accordance with the Canadian Tri-Council Policy Statement on Ethical Conduct for Research Involving Humans and the Declaration of Helsinki. All participants provided informed written consent.

#### ONDRI participants

A total of 520 participants diagnosed with one of several neurodegenerative or cerebrovascular diseases (Alzheimer’s disease, MCI, amyotrophic lateral sclerosis, FTD, Parkinson’s disease or cerebrovascular disease) were recruited from 14 tertiary care clinics in Ontario and categorized into one of five cohorts: (i) Alzheimer’s disease/MCI, (ii) amyotrophic lateral sclerosis, (iii) FTD, (iv) Parkinson’s disease or (v) cerebrovascular disease. Participant age and sex distributions by cohort are displayed in Table 1 and Supplementary Fig. 1. Inclusion/exclusion criteria and recruitment protocols for ONDRI participants have been previously reported in detail.^22,23^ All participants were diagnosed by experienced clinicians and met consensus diagnostic criteria current at the time of recruitment (detailed in Supplementary Methods).

**Table 1:** Demographic characteristics of participants included in final analyses; see also Supplementary Fig. 1

Subgroup	Number of participants (male:female)	Mean age in years (SD, range)
Control	149 (51:98)	59.4 (11.9, 42.0–87.3)
**MCI**	**68** (**36:32)**	**69.7 (8.0, 53.4–86.4)**
**Alzheimer’s disease**	**24** (**17:7)**	**72.1 (8.0, 55.9–87.8)**
*Atypical Alzheimer’s disease*	*2*	
**Amyotrophic lateral sclerosis-CN**	**14** (**10:4)**	**60.9 (7.1, 42.1–68.2)**
**Amyotrophic lateral sclerosis-CI**	**5**	
*Amyotrophic lateral sclerosis-Other*	*16* (*8:8)*	*60.0 (9.8, 40.1–72.3)*
**bvFTD**	**14** (**10:4)**	**66.6 (8.8, 49.7–77.5)**
**PSP**	**8** (**4:4)**	**70.6 (5.9, 63.5–78.5)**
*bvFTD and PSP*	*1*	
*PNFA*	*5*	
*SD*	*3*	
*CBS*	*2*	
**Parkinson’s disease-CN**	**43** (**32:11)**	**66.1 (6.3, 55.3–80.4)**
**Parkinson’s disease-MCI**	**43** (**36:7)**	**68.2 (6.3, 55.1–81.7)**
**Parkinson’s disease-dementia**	**14** (**11:3)**	**69.1 (8.4, 56.7–84.4)**
*Parkinson’s disease-Other*	*6* (*5:1)*	*73.5 (7.7, 67.6–85.9)*
**Cerebrovascular disease-CN**	**69** (**46:23)**	**68.5 (6.3, 55.0–81.4)**
**Cerebrovascular disease-MCI**	**31** (**19:12)**	**67.6 (7.4, 56.4–84.0)**
**Cerebrovascular disease-dementia**	**11** (**8:3)**	**69.0 (10.1, 57.2–85.4)**
*Cerebrovascular disease-Other*	*12* (*9:3)*	*67.1 (7.1, 58.3–83.4)*

Bolded groups were included in all analyses; italicized groups were included in factor analysis and correlations only; controls were included in parameter-level analysis only. Demographic details are not included for subgroups with ≤5 participants due to confidentiality concerns. bvFTD, behavioural variant frontotemporal dementia; CBS, corticobasal syndrome; CI, cognitively impaired; CN, cognitively normal; MCI, mild cognitive impairment; Other, other cognitive status; PNFA, progressive non-fluent aphasia; PSP, progressive supranuclear palsy; SD, semantic dementia.

All ONDRI participants completed a comprehensive test battery including neuropsychological assessment and IPAST (described in detail below). Participants also were required to enrol with a study partner who was familiar with their cognitive ability and daily functioning and whose answers on some questionnaires were used to evaluate overall cognitive status (see below). Although data were collected annually for up to 3 years, here we report only baseline data collected at each participant’s first visit.

#### Control participants

One hundred and forty-nine healthy control participants with no known neurological or psychiatric conditions were recruited from the community in Kingston, ON, Canada, as part of a larger control cohort study.^25^ All controls completed the same eye-tracking assessment as ONDRI participants, plus the Montreal Cognitive Assessment (MoCA).^26^ They did not complete other clinical or neuropsychological assessments. All controls were aged 42–87 years (the minimum and maximum ages of ONDRI participants included in parameter-level analysis; Supplementary Fig. 1) and had a normal MoCA score ≥26. We included all participants from the control cohort study whose age was comparable to any ONDRI participants to maximize the cohort size and minimize arbitrary exclusion of data; however, due to the comparatively young age of the amyotrophic lateral sclerosis cohort, the average age of the control cohort was younger than most ONDRI participants. However, we corrected all oculomotor parameters for age and sex (see ‘Calculation of saccade parameters’ section) and the inclusion of many control participants strengthened our estimation of normal behaviour throughout aging.

### Experimental protocol

#### Eye-tracking protocol

Details of the eye-tracking protocol are published^25,27^ and additionally described in Supplementary Methods. Briefly, participants completed IPAST (gap paradigm) consisting of two blocks of 120 trials approximately 7 min each in duration, plus time required for setup and calibration (30 min maximum). The colour of a central fixation point instructed participants to look at a peripheral stimulus (prosaccade) or in the opposite direction (antisaccade) (Supplementary Fig. 2). Saccade conditions (prosaccade or antisaccade) and stimulus locations (left or right) were pseudo-randomly interleaved with equal frequency. Participants were required to have normal or corrected-to-normal vision in at least one eye and were excluded if they had visual field defects obscuring targets within ±10 degrees of central vision.

#### Clinical assessment

All ONDRI participants were recruited by specialist physicians and enrolled in one of five cohorts according to consensus diagnostic criteria. Two cohorts were further divided into subgroups based on diagnosis: Alzheimer’s disease/MCI was subdivided into Alzheimer’s disease, MCI or atypical Alzheimer’s disease; FTD was subdivided into bvFTD, PSP, progressive non-fluent aphasia (PNFA), semantic dementia (SD) or corticobasal syndrome (CBS). These subgroups were used for subsequent analysis in this study. The remaining three cohorts (amyotrophic lateral sclerosis, Parkinson’s disease and cerebrovascular disease) were not subdivided by diagnostic criteria but were subdivided based on neuropsychological assessment of overall cognitive status (details below). Note that the FTD cohort alone was not subdivided using an approach related to overall cognitive impairment; however, we considered the diagnostic subgroups highly relevant due to their clinical applicability.

#### Neuropsychological assessment

ONDRI participants completed a comprehensive neuropsychological test battery.^22,24^ The results of these tests were used to (i) classify cognitive status in cohorts without subgroups (i.e. amyotrophic lateral sclerosis, Parkinson’s disease, and cerebrovascular disease) and (ii) calculate cognitive domain scores for all ONDRI participants across five cognitive domains (attention/working memory, executive function, language, memory, and visuospatial function, as described elsewhere).^28^ Cognitive status was determined using the neuropsychological test scores, subjective cognitive decline questionnaires (completed by participants and study partners), and activities of daily living (completed by study partners). Cognitive domain scores were calculated using 23 neuropsychological test scores divided into the five domains. See Supplementary Methods and Supplementary Table 1 for details of both calculations. All resultant subgroups used in this study are shown in Table 1.

### Data analysis

#### Saccade classification

Preprocessing of saccade data is described in detail in the Supplementary Methods and Coe *et al.*^27^ Trials with lost tracking, without saccade behaviour, or with other aberrations [e.g. very slow reaction time (>800 ms), saccades to random locations and failure to fixate centrally during the fixation period] were excluded; 9834 of 145 990 trials (6.74%) were excluded across the 485 ONDRI and 149 control participants who successfully completed eye-tracking assessment.

Saccades were subsequently classified based on their timing relative to specific task events (Supplementary Fig. 2). Saccadic reaction time (SRT) was defined as the time between the appearance of the peripheral stimulus and saccade initiation; only the first saccade in each trial was considered for analysis.

Saccade classification accounted for the minimum 90 ms delay for afferent visual signals to trigger a saccade.^29^ Saccades of reaction time 90–139 ms after stimulus appearance were considered express latency saccades, and those occurring 140–800 ms were considered regular latency saccades. Express and regular latency saccades were also separated based on correctness. Saccades initiated 110 ms before to 89 ms after stimulus appearance indicated guessing behaviour, since they occurred while the participant perceived a blank screen, and were classified as anticipatory saccades. Task disengagements were classified as trials where the participant looked away from the fixation point (1110 ms before to 111 ms before stimulus appearance) and did not re-fixate it. SRT (ms) was measured for initial prosaccades and antisaccades; additionally, peak velocity (degrees/s) and amplitude (degrees) were measured for correct prosaccades.

#### Calculation of saccade parameters

The 12 parameters of interest (details in Supplementary Methods and Supplementary Table 2) were selected to represent key behaviours occurring throughout the task. We calculated the following: percentages of prosaccade and antisaccade task disengagements, percentages of anticipatory prosaccades and antisaccades, mean prosaccade and antisaccade SRT, percentage of express latency correct prosaccades, percentages of express latency and regular latency antisaccade direction errors, voluntary override time (VOT), and mean correct prosaccade velocity and amplitude. These measures were chosen to robustly sample various aspects of participants’ oculomotor behaviour, as they encompass both tasks and the entire time course of each trial. Notably, VOT is a relatively novel parameter that indicates the time when voluntary antisaccade-generating processes begin to override automated prosaccade-generating processes^25^ and has not been characterized in previous studies of neurodegenerative disease.

All 12 parameters were then age- and sex-corrected to remove any effects of these variables between groups, including between the control group and ONDRI subgroups. A linear regression was fit to the control data alone for each measure with age and sex as predictors; most previously reported parameters follow a generally linear trend over the age range included in this study.^25^ The resulting regression equation, whose output represented the expected value of each parameter for a healthy control given their age and sex, was then used to calculate standardized residual values on each parameter for each participant, including ONDRI participants. These standardized residuals were then used in all subsequent analyses.

#### Study-specific quality control and exclusion criteria

Of the 520 recruited ONDRI participants, 485 successfully completed eye-tracking assessment (Supplementary Fig. 3A and B). We then applied several additional criteria for data quality control to exclude participants with poor data. This resulted in 391 ONDRI participants included in the final factor analysis and subsequent correlations. Note that control participants were excluded from factor analysis as they did not complete neuropsychological testing and were therefore ineligible for subsequent analyses correlating factor scores to neuropsychology-based cognitive domain scores. We additionally removed small subgroups [*n* ≤ 5, except for amyotrophic lateral sclerosis-cognitively impaired (CI) that was retained for comparison to amyotrophic lateral sclerosis cognitively normal (CN)] and participants with cognitive status ‘other’ for parameter-level analysis. Three hundred and forty-four ONDRI participants (across 12 subgroups) and 149 control participants were included in the final parameter-level analysis for most oculomotor parameters. For additional detail regarding final subgroup membership and exclusion criteria, see Table 1, Supplementary Fig. 3 and Supplementary Methods.

#### Statistical analysis

All statistical analyses were performed using SPSS version 27 (IBM, Armonk, NY, USA). Deviations from normality were assessed by visual inspection and verified by Shapiro–Wilk tests.

Exploratory factor analysis was conducted on 12 oculomotor parameters using principal axis factoring and oblique rotation (direct oblimin). These were chosen based on recommendations that principal axis factoring be used for extraction on non-normally distributed data and that oblique rotation be used consistently given its ability to produce accurate solutions if factors are correlated and to reproduce orthogonal solutions if factors are not correlated.^30^ Factor loadings were considered significant at an absolute value ≥0.3.^31^

Factor scores were calculated for each participant using the regression method, and Spearman’s correlations were subsequently used to evaluate the relationships between the four factors and five neuropsychology composite domain scores. A Holm–Bonferroni correction was applied to the 20 correlations to adjust for multiple comparisons. Correlations were considered significant at adjusted *P* ≤ 0.05 (two-tailed) and are reported in adjusted format.

Kruskal–Wallis tests were conducted to compare parameters between disease subgroups and controls. Significant findings were followed by *post hoc* two-sided Dunn’s tests to investigate individual pairwise comparisons. Only pairwise comparisons between the control group and each disease subgroup, as well as between subgroups within each cohort, were conducted; although different approaches were used to subdivide the cohorts by diagnosis or cognitive status, no subgroups with different categorization systems were directly compared. The Holm–Bonferroni correction was applied to adjust for multiple comparisons. Kruskal–Wallis tests were considered significant at an alpha level of *P* = 0.05; *post hoc P*-values were considered significant at adjusted *P* = 0.05 and are reported in adjusted format.

## Results

### Factor analysis

**Figure 1:**
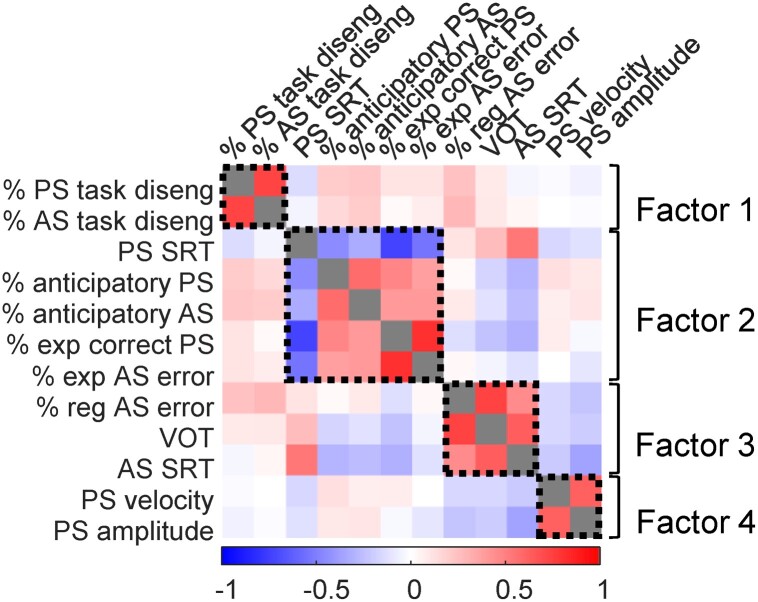
**Correlations between 12 IPAST measures of interest and underlying factor structure.** Correlations include all ONDRI participants included in factor analysis (*N* = 391); variables are age- and sex-corrected standardized residuals. Colour bar indicates Pearson correlation coefficients between pairs of variables; grey squares indicate correlation coefficients of 1. Black dashed boxes indicate groups of variables that loaded together on each factor displayed on the right. AS, antisaccade; PS, prosaccade; SRT, saccadic reaction time; VOT, voluntary override time.

We constructed a correlation matrix with all 12 age- and sex-corrected IPAST parameters, including all ONDRI participants (Fig. 1) to determine whether and how these parameters were related. We hypothesized that measures related to similar cognitive or motor processes (e.g. anticipatory saccades and express latency antisaccade errors, which could both indicate impulsivity or lack of inhibitory control; prosaccade velocity and amplitude, which are both fundamental motor characteristics) might correlate. Moderate to high correlations were present between some parameters (Fig. 1). We therefore performed an exploratory factor analysis on the ONDRI data to characterize these associations and their possible underlying factors.

The Kaiser–Meyer–Olkin (KMO) measure, which indicates the proportion of potentially common variance among parameters and therefore the suitability of the data for factor analysis, demonstrated appropriate sampling adequacy (*KMO* = 0.67), and all KMO values for individual variables were above 0.5. Bartlett’s test of sphericity (*χ*^2^(66) = 2321.77, *P* < 0.001) indicated large enough correlations between variables to perform factor analysis. Four factors had eigenvalues above Kaiser’s criterion of 1 and explained 64.04% of the variance prior to rotation. Visual inspection of the Scree plot was ambiguous and did not display an obvious inflexion point; given the relatively small number of variables, large sample size, and average communality following extraction >0.6, Kaiser’s criterion was considered suitable and four factors were extracted in the final solution, each of which we hypothesized might characterize different underlying circuitry and cognitive processes. Factor structure following rotation is displayed in Fig. 1, Table 2 and Supplementary Table 3.

**Table 2:** Pattern matrix for factor analysis

IPAST parameter	Factor 1	Factor 2	Factor 3	Factor 4
Prosaccade task disengagements	**−0**.**85**	0.03	0.01	−0.07
Antisaccade task disengagements	**−0**.**84**	−0.05	0.03	−0.009
Prosaccade SRT	−0.01	**−0**.**72**	0.14	−0.07
Anticipatory prosaccades	−0.15	**0**.**54**	−0.04	0.09
Anticipatory antisaccades	−0.20	**0**.**47**	0.008	0.08
Express correct prosaccades	0.14	**0**.**96**	−0.04	−0.09
Express antisaccade errors	0.11	**0**.**84**	0.12	−0.12
Regular antisaccade errors	−0.17	0.09	**0**.**74**	0.001
Voluntary override time (VOT)	0.08	0.001	**1**.**0**	0.09
Antisaccade SRT	0.08	−0.23	**0**.**58**	−0.19
Prosaccade velocity	0.03	0.03	0.03	**0**.**62**
Prosaccade amplitude	0.04	−0.10	−0.02	**0**.**99**

*N* = 391; extraction method: principal axis factoring; rotation method: oblique (direct oblimin with Kaiser normalization). Factor loadings above 0.3 are displayed in bold.

Prosaccade and antisaccade task disengagements loaded together on factor 1, which we considered to be a potential measure of attentional control or task-relevant motivation. Prosaccade SRT, anticipatory prosaccades and antisaccades, express correct prosaccades, and express antisaccade errors loaded together on Factor 2, which appeared related to the integrity of global inhibitory processes and impulsivity. Antisaccade SRT, regular antisaccade errors, and VOT loaded on Factor 3, suggesting it might be related to cognitive impairment or voluntary saccade generation. Prosaccade velocity and amplitude loaded on Factor 4, which likely represented the integrity of brainstem circuits controlling saccade metrics and dynamics.

Although Factors 1 and 4 were composed of only two variables each, the conceptual and biological links between each pair of variables were such that we considered the factors valid. Additionally, most of the variables concerned loaded strongly on their respective factors, and the large sample size of the study reinforces the stability of the factors.^32^

We then performed follow-up analyses related to each factor. First, we generated correlations between factor scores and neuropsychology-based cognitive domain scores. Second, we performed between-group comparisons of the individual component parameters from each factor to explore differences between disease subgroups and controls who were not included in the factor analysis.

### Cognitive domain analysis

**Figure 2:**
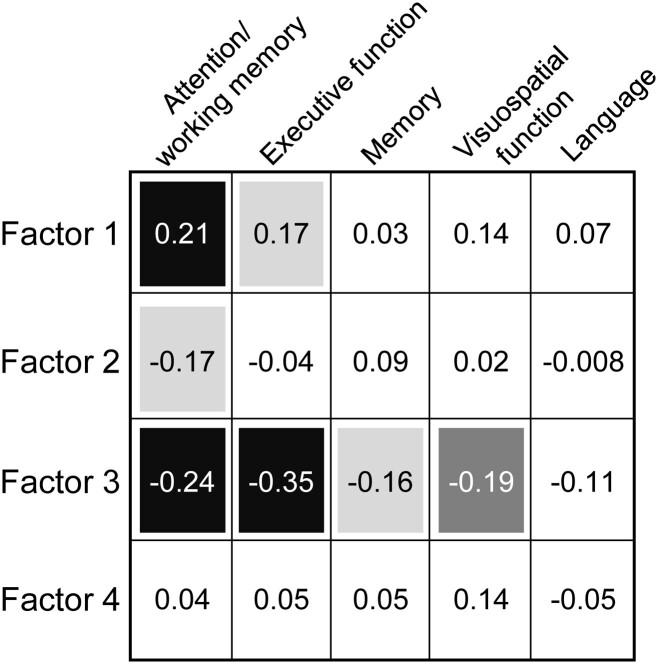
**Correlations between factor scores and neuropsychology cognitive domain scores.** Correlations include all ONDRI participants included in factor analysis (*N* = 391). Shaded boxes indicate significant correlations: light grey, *P* ≤ 0.05; dark grey, *P* ≤ 0.01; black, *P* ≤ 0.001 (all *P* Holm–Bonferroni adjusted). Values inside boxes indicate Spearman’s rho.

Correlations were conducted to characterize the relationships between robust, aggregate measures of oculomotor and cognitive function. We hypothesized that each oculomotor factor might correlate with cognitive domains ostensibly related to its component variables. Figure 2 displays correlation results.

Factor 1 (task disengagements) scores were positively correlated with scores of attention/working memory (*r* = 0.21, *P* < 0.001) and executive function (*r* = 0.17, *P* = 0.011). Factor 2 (prosaccade SRT, anticipatory prosaccades and antisaccades, express correct prosaccades and express antisaccade errors) scores were significantly correlated only with attention/working memory (*r* = −0.17, *P* = 0.016). Factor 3 (antisaccade SRT, regular antisaccade errors and VOT) scores were significantly negatively correlated with four of five cognitive domain scores: attention/working memory (*r* = −0.24, *P* < 0.001), executive function (*r* = −0.35, *P* < 0.001), memory (*r* = −0.16, *P* = 0.017), and visuospatial function (*r* = −0.19, *P* = 0.0021). Finally, Factor 4 (saccade amplitude and velocity) correlated with no cognitive domain scores. No oculomotor factors correlated with language scores.

### Parameter-level analysis

Between-group analysis was performed to quantify differences on each of the 12 age- and sex-corrected parameters of interest (i.e. standardized residuals) defined in ‘Methods’ section. This provided a direct point of comparison to existing prosaccade and antisaccade literature in neurodegenerative and cerebrovascular disease and allowed us to describe disease-related behavioural alterations. All reported *post hoc P*-values were adjusted for multiple comparisons. Here, we report a subset of six parameters in which between-group differences were of particular interest; the remaining parameters are reported in Supplementary Results and Supplementary Figs 4 and 5.

#### Task disengagements

**Figure 3:**
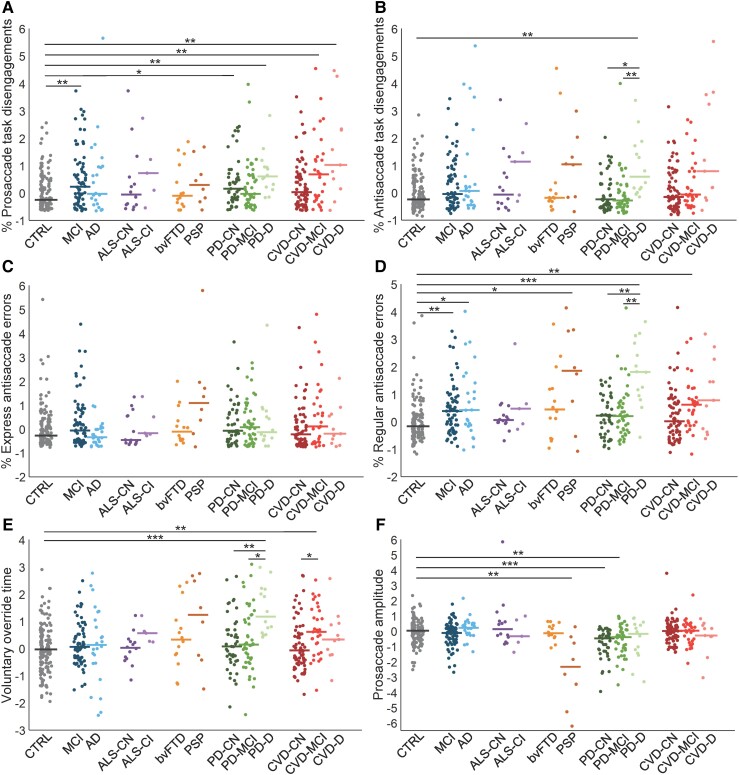
**Age- and sex-corrected eye-tracking measures by subgroup.** (**A**) Standardized residual of percentage of prosaccade task disengagements; (**B**) standardized residual of percentage of antisaccade task disengagements; (**C**) standardized residual of percentage of express latency antisaccade errors; (**D**) standardized residual of percentage of regular latency antisaccade errors; (**E**) standardized residual of voluntary override time; (**F**) standardized residual of prosaccade amplitude. Each point represents a single participant; horizontal lines show subgroup medians. *P < 0.05; **P < 0.01; ***P < 0.001 (Kruskal–Wallis tests followed by *post hoc* Dunn’s tests with Holm–Bonferroni correction for multiple comparisons). AD, Alzheimer’s disease; ALS, amyotrophic lateral sclerosis; bvFTD, behavioural variant frontotemporal dementia; CI, cognitively impaired; CN, cognitively normal; CTRL, control; CVD, cerebrovascular disease; D, dementia; MCI, mild cognitive impairment; PD, Parkinson’s disease; PSP, progressive supranuclear palsy.

Task disengagements (shifting gaze away from the fixation point displayed onscreen and failing to return to it) may indicate poor attentional control or lack of motivation; these parameters loaded on Factor 1. A Kruskal–Wallis test revealed significant differences between groups in prosaccade task disengagements (*H*(12) = 40.77, *P* < 0.001) and antisaccade task disengagements (*H*(12) = 37.43, *P* < 0.001) (Fig. 3A and B). *Post hoc* testing indicated that the MCI (*z* = 3.72, *P* = 0.0042), Parkinson’s disease CN (*z* = 3.05, *P* = 0.039), Parkinson’s disease-dementia (*z* = 3.61, *P* = 0.0058), cerebrovascular disease-MCI (*z* = 3.70, *P* = 0.0043), and cerebrovascular disease-dementia (*z* = 3.51, *P* = 0.0081) groups made more prosaccade task disengagements than the control group. The Parkinson’s disease-dementia (*z* = 3.74, *P* = 0.0037) group also made significantly more antisaccade task disengagements relative to controls. There were additional significant differences between subgroups within the Parkinson’s disease cohort; the Parkinson’s disease-dementia group made more antisaccade task disengagements than both the Parkinson’s disease-CN (*z* = 3.37, *P* = 0.015) and Parkinson’s disease-MCI (*z* = 3.87, *P* = 0.0023) groups. In the amyotrophic lateral sclerosis and cerebrovascular disease cohorts, task disengagements also appeared higher in more cognitively impairedsubgroups, although these comparisons were not statistically significant.

#### Express latency antisaccade errors

We tested for between-group differences in express latency antisaccade errors, which loaded on Factor 2. Prosaccades at this latency typically reflect the integrity of visuomotor circuitry that generates a visually driven saccade, whereas antisaccade errors (i.e. inappropriate prosaccades) reflect failure of pre-emptive inhibitory control mechanisms to suppress signals propagating through this circuitry. There were no differences between groups in express latency antisaccade errors (*H*(12) = 19.18, *P* = 0.084) (Fig. 3C).

#### Regular latency antisaccade errors

While express latency errors (loaded on Factor 2) denote failure of pre-emptive inhibitory processes, regular latency antisaccade errors (loaded on Factor 3) denote failure of voluntary saccade generation signals that programme a saccade away from the stimulus to outcompete automated cortical signals that programme a saccade to the stimulus. There were significant differences between groups in regular antisaccade errors (*H*(12) = 57.52, *P* < 0.001) (Fig. 3D). *Post hoc* tests showed significantly higher proportions of regular latency errors in the MCI (*z* = 3.67, *P* = 0.0044), Alzheimer’s disease (*z* = 2.96, *P* = 0.046), PSP (*z* = 3.02, *P* = 0.040), Parkinson’s disease-dementia (*z* = 5.43, *P* < 0.001), and cerebrovascular disease-MCI (*z* = 4.01, *P* = 0.0012) groups relative to the control group. No cognitively normal subgroups were different from controls (all *P* > 0.05). Within cohorts, Parkinson’s disease-dementia made significantly more errors compared to both Parkinson’s disease-CN (*z* = 3.79, *P* = 0.0029) and Parkinson’s disease-MCI (*z* = 3.59, *P* = 0.0055). Although not statistically significant, the median value of amyotrophic lateral sclerosis-CI was higher than that of amyotrophic lateral sclerosis-CN, and the median values of cerebrovascular disease-MCI and cerebrovascular disease-dementia were higher than cerebrovascular disease-CN.

#### Voluntary override time

This measure, which also loaded onto Factor 3, indicates the time during antisaccade trials at which voluntary signals begin to outcompete automated signals to produce correct antisaccades.^33^ Significant between-group differences were also revealed in VOT (*H*(12) = 40.77, *P* < 0.001) (Fig. 3E), with the Parkinson’s disease-dementia (*z* = 4.80, *P* < 0.001) and cerebrovascular disease-MCI (*z* = 3.53, *P* = 0.0078) groups showing significantly slower VOTs than the control group. Within cohorts, Parkinson’s disease-dementia displayed slower VOT than Parkinson’s disease-CN (*z* = 3.72, *P* = 0.0039) and Parkinson’s disease-MCI (*z* = 3.45, *P* = 0.010); cerebrovascular disease-MCI displayed slower VOT than cerebrovascular disease-CN (*z* = 3.16, *P* = 0.027). Some within-cohort comparisons (amyotrophic lateral sclerosis-CN versus amyotrophic lateral sclerosis-CI; cerebrovascular disease-dementia versus cerebrovascular disease-CN) also appeared to indicate slower VOT in cognitively impaired subgroups but did not reach statistical significance.

#### Saccade amplitude

Alterations in saccade metrics such as amplitude, which loaded on Factor 4, may indicate brainstem dysfunction due to its fundamental role in saccade generation. Significant differences between groups were indicated for prosaccade amplitude (*H*(12) = 53.05, *P* < 0.001) (Fig. 3F). *Post hoc* tests revealed smaller prosaccade amplitude in the PSP (*z* = 3.72, *P* = 0.0040), Parkinson’s disease-CN (*z* = 5.13, *P* < 0.001), and Parkinson’s disease-MCI (*z* = 3.51, *P* = 0.0086) groups compared to controls.

## Discussion

Via factor analysis and cognitive domain scores, we elucidated the factor structure underlying prosaccade and antisaccade measures in neurodegeneration and correlated these factors to several cognitive domains. We also examined behavioural differences between disease groups and healthy controls. Collectively, these results suggest that several dissociable brain processes underlie related subsets of IPAST measures, that these processes differentially contribute to distinct cognitive functions, and that IPAST identifies cognitive impairment in neurodegenerative and cerebrovascular disease.

### Neural and cognitive correlates of IPAST behaviour

Our approach combined multiple IPAST parameters and neuropsychological test scores to produce robust characterizations of participants’ oculomotor behaviour and cognition, respectively. Previous work has largely reported bivariate correlations of individual saccade parameters and neuropsychological test scores,^34–36^ with a very small minority performing principal component analysis.^37^ Factor analysis of the interrelationships between IPAST parameters generated four factors (Fig. 1), which we propose correspond to four functionally distinct neural processes. In turn, heterogeneous disease-related dysfunction in these processes and their underlying circuitry may differentially affect oculomotor behaviour, affording potential sources of behavioural biomarkers for neurodegenerative diagnoses and/or their clinical characteristics. Our subsequent analysis of the relationships between IPAST factors and cognitive domain scores revealed associations of these factors to measured cognitive domains (attention/working memory, executive function, memory, and visuospatial function).

Prosaccade and antisaccade task disengagements (looking away from fixation point without returning) loaded on Factor 1. This suggests that Factor 1 relates to processes governing task engagement or motivation that must be maintained during the fixation epoch for successful task performance. Interpretation of Factor 1 as measuring sustained attentional or motivational control is supported by its correlation with attention/working memory scores, although it also correlated with executive function scores.

Several IPAST parameters loaded on Factor 2 (prosaccade SRT, anticipatory prosaccade/antisaccade, express latency correct prosaccades and express latency antisaccade errors; i.e. parameters occurring at very short latencies). Following stimulus appearance, visual signals propagate through retino–geniculo–cortical–tectal and retinotectal pathways to the superior colliculus (SC), where they can produce short-latency visually triggered express saccades.^38,39^ These visual signals also pass from visual cortex to parietal regions such as the parietal eye fields (PEF), which generate automated saccades to visual stimuli via projections to SC.^29,40^ Factor 2 may therefore index either the integrity of these processes or the global suppression of automated visual signals. Attention/working memory was the only cognitive domain correlated with Factor 2. However, the lack of other significant associations suggests that neural processes comprising Factor 2 may have a limited impact on cognition; the short latencies of its component parameters suggest some relationship to response inhibition, which requires sustained task-directed attentional control, but not to complex executive functions that typically take longer to come online. Previous findings regarding the constituent parameters are mixed, with correlations found to various cognitive domains^41,42^ or to none.^36,43^ Factor 2 is therefore unlikely to be a meaningful indicator of widespread cognitive dysfunction in neurodegenerative or cerebrovascular disease but may provide an index of attentional or inhibitory processes.

The best-understood and most-reported antisaccade parameters are antisaccade errors and antisaccade latency. These loaded together with VOT on Factor 3 (note that regular latency errors loaded on Factor 3, while express latency errors loaded on Factor 2, indicating that they index disparate neural processes; see below). Antisaccade errors, which in most studies are not separated by latency, are overwhelmingly linked to dysfunction in frontal regions such as the frontal eye fields (FEF), supplementary eye fields (SEF), and dorsolateral prefrontal cortex (DLPFC). Along with the basal ganglia, these regions are required to pre-emptively inhibit automated signals driving an erroneous visually guided prosaccade and instead generate a voluntary antisaccade in the opposite (correct) direction.^15,16^ Damage to such frontal regions increases error production^16,44,45^ and may slow correct antisaccade latency,^44,46^ and their blood oxygen level–dependent (BOLD) signal is greater while preparing for antisaccade compared to prosaccade trials.^17,47–49^ These regions project directly to the SC and indirectly via the basal ganglia to modulate saccade behaviour. Factor 3 is therefore likely to correspond to this frontostriatal network and measure its inhibitory capacity and ability to generate voluntary task-directed movement—in particular, to generate a voluntary saccade to a location void of visual stimulus. Accordingly, it correlated with several cognitive domains related to frontal integrity (e.g. attention/working memory, executive function and memory)^50^ and spatial awareness (e.g. visuospatial function). This aligned with previous findings in neurodegenerative disease connecting antisaccade errors and/or latency to numerous cognitive domains, including attention/working memory,^36,41,51^ visuospatial function,^21,34,41^ memory^34,41,51^ and language.^21,34,51^ Previous studies often used miscellaneous neuropsychological tests to generate inconsistent patterns of antisaccade error association with cognitive domains; our findings clarify that antisaccade errors, SRT, and VOT are indeed predictive of several cognitive domains, most strongly executive function, and are therefore an appropriate behavioural biomarker for cognitive impairment and particularly dysexecutive syndromes.

Factor 4 was composed of basic saccade metrics (amplitude and velocity) and likely reflected comparatively low-level saccade generation processes occurring in the brainstem.^10^ No cognitive domain scores correlated to Factor 4, which was expected given that cortical regions modulating cognitive processes have limited involvement in controlling saccade dynamics.

Overall, the factor structure and patterns of association between factors and cognitive domains indicate that subsets of IPAST parameters differentially assess the integrity of multiple neural and cognitive processes. Neurodegenerative and cerebrovascular disease cohorts may represent particularly valuable opportunities to characterize these relationships since they comprise greater cognitive and behavioural variability than healthy controls.

### Relation to previous oculomotor studies

There is a rich tradition of antisaccade analysis in neurodegenerative disease cohorts, and our results variously corroborate, contradict or expand on existing literature. We discuss only our most interesting results here, but additional comparisons to previous studies are provided in the Supplementary Discussion.

#### Express versus regular latency errors

Importantly, we separated antisaccade errors into express and regular latency errors, which most previous studies combine. These two parameters loaded on different factors, substantiating previous work showing little correlation between them in healthy controls and an association of regular but not express latency antisaccade errors with executive function.^25,37^ Different causal processes are related to each error type: express errors result from failure of pre-emptive inhibitory processes to suppress reflexive motor output from the SC,^52^ whereas regular errors occur due to inability of voluntary saccadic processes derived from frontal cortex and basal ganglia to outcompete automated parietal signals driving a saccade to stimulus.^29^ This dissociation has been replicated by a computational model of IPAST behaviour.^33^

Supporting this, we also found a striking difference between these two types of errors at the individual parameter level, with many disease subgroups committing more regular latency antisaccade errors than controls but none displaying any differences in the occurrence of express latency errors. This implies that in neurodegenerative disease, the circuitry suppressing express latency errors remains intact or that compensatory mechanisms can offset any damage, while circuitry suppressing regular latency errors is more susceptible or cannot be adequately compensated. Interestingly, the most comparable previous reports showed increased short-latency errors in neurodegeneration^42,53^ although sample sizes were smaller, participant medication status and disease stage were dissimilar, and parameter definition was different from this study. Other studies reporting express latency antisaccades (presumably consisting primarily of express latency errors, given the scarcity of correct antisaccades at express latencies) found no effect of disease in cohorts of 35–40 participants.^54,55^ Our study corroborates and expands on these findings by more narrowly delineating the relevant parameter and comprising more participants and trials.

Our results highlight that pooling express and regular latency errors, as is done in most antisaccade studies of neurodegeneration, obscures crucial subtleties in dysfunction that can be revealed if these parameters are separated; future studies should assess these error types independently to accurately illustrate the nature of disease-related impairment.

#### Regular latency errors and overall cognitive impairment

Relatively few previous studies have investigated antisaccade behaviour in neurodegenerative disease cohorts with differing levels of cognitive impairment. Here, we determined overall cognitive impairment in disease cohorts using a robust categorization method based on neuropsychological testing. Regular latency antisaccade errors, VOT, and task disengagements scaled with cognitive impairment, suggesting that overall cognitive impairment modulates these parameters in neurodegenerative disease and that IPAST can supplement robust but time-consuming neuropsychological assessment.

We found significant differences in regular latency errors between levels of cognitive impairment in Parkinson’s disease. Although most antisaccade studies of Parkinson’s disease exclude participants with concomitant dementia and find increased error rates in patients,^13^ a small number included them and found significant results^56^ or trends^57^ indicating control-like performance in cognitively normal participants but increased errors in cognitively impaired participants, in accordance with our results. Notably, the latter study used only the MoCA to screen for cognitive impairment, whereas the former and our study both used more comprehensive neuropsychological testing, signifying the importance of robust cognitive assessment (versus cognitive screening) and implying that antisaccade errors may measure similar constructs. Future studies should continue to assess the effect of cognitive impairment on antisaccade behaviour in other neurodegenerative and cerebrovascular diseases.

#### Task disengagements

Task disengagements (departures of gaze from the fixation point without returning) may also scale with cognitive impairment. Generally, cognitively impaired subgroups made more task disengagements than controls. Previous studies have found fixation abnormalities (e.g. square-wave jerks and other involuntary saccadic intrusions) in various neurodegenerative diseases;^11^ however, task disengagements in this study do not represent involuntary saccadic intrusions because participants successfully performed subsequent task-irrelevant fixation. It is unclear whether initial fixation disengagement was voluntary or involuntary, but failure to re-fixate suggests the exclusive or additional involvement of voluntary processes. Reports of identical^53^ or similar^58,59^ parameters similarly indicate increased frequency in neurological or psychiatric disorders, but understanding of their neural and cognitive correlates is severely limited by the paucity of studies using them as an outcome measure. Future work should probe the dysfunction that may augment task disengagement frequency in cognitively impaired individuals.

#### Movement metrics

IPAST can also evaluate oculomotor dysfunction in movement disorders such as Parkinson’s disease and PSP, making it a useful screening tool for both motor and cognitive deficits. We found that both Parkinson’s disease-CN and Parkinson’s disease-MCI displayed hypometric prosaccades relative to controls, as in many previous studies,^53,60,61^ although we did not observe significant hypometria in Parkinson’s disease-dementia. We additionally observed reduced saccade amplitude in PSP in accordance with existing reports.^11,62,63^ These were the only disorders to show differences in saccade metrics, so IPAST may serve as a detector of Parkinsonian motor dysfunction in addition to cognitive impairment.

### Limitations and future directions

We did not consider clinical variables such as disease severity and medication. Additionally, we employed a conservative statistical approach to investigate between-group differences. Future cohort- or subgroup-specific studies should investigate the oculomotor effects of medication type, dosage, and other clinical parameters, and the statistical challenges produced by analysing many heterogeneous groups would be less applicable to smaller-scale designs.

This study is among a very small number to report prosaccade and antisaccade behaviour in cerebrovascular disease or related diseases. Behaviour in this cohort is heterogeneous; as demonstrated by lesion studies, stroke location determines the resultant behavioural deficits, but we considered location-based analysis too complex for this study. Accordingly, the cerebrovascular disease cohort is best considered an illustration of our findings about cognitive impairment. Some participants could have performed poorly due to insufficient visual acuity or oculomotor ability resulting from localized lesions. However, cerebrovascular disease collectively did not display prosaccade deficits, indicating that visuomotor circuitry was largely intact. Stroke location should be considered in future studies.

Finally, although only baseline (first visit) data were reported in the current study, ONDRI participants were assessed longitudinally. Evaluation of changes in oculomotor behaviour over time due to cognitive decline should be completed as a supplement to our results.

## Conclusions

In summary, we used factor analysis to define a novel framework for the interrelationships of IPAST parameters in the ONDRI cohort and propose underlying neural correlates for each factor, collectively comprising a diffuse network of cortical and subcortical regions that encompasses much of the brain; these factors may therefore have great power to detect neurodegenerative processes regardless of location. Next, we examined correlations of IPAST factors to cognitive domains. Regular latency antisaccade errors appeared related to cognitive impairment and dissociated from express latency errors; VOT and task disengagements also increased with cognitive impairment. Most subgroups did not display motor deficits, with the predictable exceptions of PSP and Parkinson’s disease. This suggests that IPAST is sensitive to cognitive impairment across diseases and provides additional information regarding movement abnormalities in some diseases. Collectively, these relationships accentuate the ability of IPAST to output behavioural measures that evaluate distinct cognitive functions and their neural substrates and support its use to assess cognitive dysfunction in neurodegenerative and cerebrovascular disease. Although it requires specialized equipment and trained operators, and may present some physical challenges for elderly participants, oculomotor assessment is more time- and cost-effective than other means of investigation such as brain imaging and neuropsychological assessment. Therefore, this work could form the foundation of a screening tool using eye movements to evaluate both motor and cognitive dysfunction in neurodegenerative disease states. In future, such a tool might also prove beneficial in the detection and characterization of prodromal states.

## Supplementary Material

fcad049_Supplementary_Data

## Data Availability

All baseline data collected during the ONDRI foundational study, including all data supporting the findings of this study, are available on request from the Ontario Brain Institute (OBI) (details at www.ondri.ca).

## Appendix I

ONDRI Investigators: Sabrina Adamo, Rob Bartha, Courtney Berezuk, Alanna Black, Michael Borrie, Susan Bronskill, Dennis Bulman, Leanne Casaubon, Ben Cornish, Sherif Defrawy, Allison Dilliott, Roger A. Dixon, Sali Farhan, Frederico Faria, Julia Fraser, Morris Freedman, Mahdi Ghani, Barry Greenberg, Hassan Haddad, Ayman Hassan, Wendy Hatch, Rob Hegele, Melissa Holmes, Chris Hudson, Mandar Jog, Peter Kleinstiver, Donna Kwan, Elena Leontieva, Brian Levine, Efrem Mandelcorn, Ed Margolin, Bill McIlroy, Manuel Montero-Odasso, David Munoz, Nuwan Nanayakkara, Miracle Ozzoude, Joel Ramirez, Natalie Rashkovan, John Robinson, Ekaterina Rogaeva, Yanina Sarquis Adamson, Christopher Scott, Michael Strong, Sujeevini Sujanthan, Sean Symons, Athena Theyers, Angela Troyer, Karen Van Ooteghem, John Woulfe and Mojdeh Zamyadi.

## Abbreviations

bvFTD =: behavioural variant frontotemporal dementia
CBS =: corticobasal syndrome
CI =: cognitively impaired
CN =: cognitively normal
DLPFC =: dorsolateral prefrontal cortex
FEF =: frontal eye fields
FTD =: frontotemporal dementia
IPAST =: interleaved prosaccade and antisaccade task
IQR =: interquartile range
MCI =: mild cognitive impairment
MoCA =: Montreal Cognitive Assessment
ONDRI =: Ontario Neurodegenerative Disease Research Initiative
PEF =: parietal eye fields
PNFA =: progressive non-fluent aphasia
PSP =: progressive supranuclear palsy
SC =: superior colliculus
SD =: semantic dementia
SEF =: supplementary eye fields
SRT =: saccadic reaction time
VOT =: voluntary override time
