# Supplementary material for: Cognitive correlates of antisaccade behaviour across multiple neurodegenerative diseases
Source: Brain Commun. 2023 Mar 2;5(2):fcad049. doi: 10.1093/braincomms/fcad049 (PMC10036290; doi:10.1093/braincomms/fcad049)
Supplement: fcad049_Supplementary_Data [file fcad049_supplementary_data.pdf]

Supplementary Material

This document contains supplementary material for "Cognitive correlates of antisaccade behaviour across multiple neurodegenerative diseases" by Riek *et al*. Herein we describe:

1) additional and more detailed methodology (participant demographics, oculomotor recording specifications, detailed calculations of oculomotor and neuropsychological parameters, exclusion criteria and quality control procedures)
2) additional results (structure matrix from factor analysis, cumulative saccade distributions, and parameter-level results from six parameters not included in main text)
3) additional discussion (comparisons to previous disease-specific studies, limitations and future directions)

Supplementary Methods

Participant age and sex

Participant age and sex distributions are displayed below in Supplementary Fig. 1. Note that subgroups containing ≤5 participants are not shown due to confidentiality concerns.

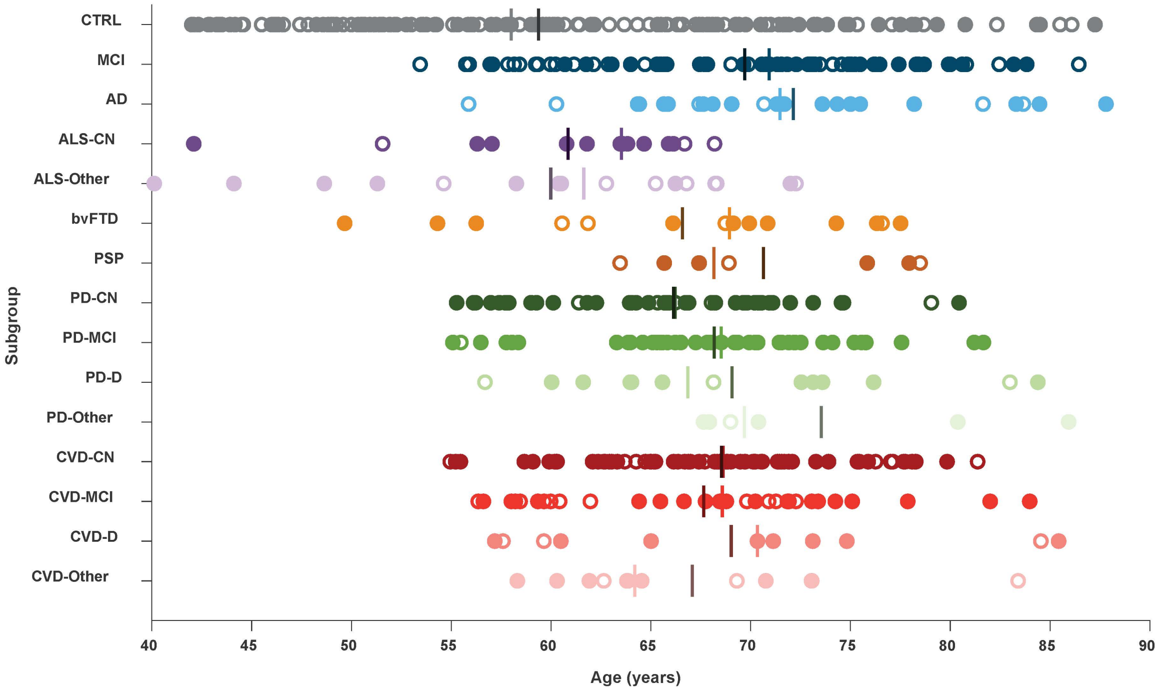

Supplementary Figure 1. Age distribution of all subgroups containing ≥5 participants. Each circle represents the age of one male participant and each cross represents the age of one female participant. Dark and light vertical lines represent mean and median ages, respectively, of each group. Control participants were included only in parameter-level analysis; participants with "Other" cognitive status were included only in factor analysis; all other participants were included in both analyses. Atypical AD, ALS-CI, PNFA, SD, CBS, and bvFTD/PSP participants are not shown due to confidentiality issues related to the small size of these subgroups, but were included only in factor analysis. CTRL: control. MCI: mild cognitive impairment. AD: Alzheimer's disease. ALS: amyotrophic lateral sclerosis. bvFTD: behavioural variant frontotemporal dementia. PSP: progressive supranuclear palsy. CBS: corticobasal syndrome. PNFA: progressive nonfluent aphasia. SD: semantic dementia. PD: Parkinson's disease. CVD: cerebrovascular disease. CN: cognitively normal. CI: cognitively impaired. D: dementia. Other: cognitive status unclear.

Cohort diagnostic criteria

The specific diagnostic criteria used for each ONDRI cohort was as follows: the National Institute on Aging-Alzheimer's Association criteria for probable Alzheimer's disease dementia or amnestic MCI[1,2]; possible, probable, or definite ALS based on El Escorial criteria[3]; subtype criteria for behavioural variant frontotemporal dementia (bvFTD),[4] progressive supranuclear palsy (PSP),[5] corticobasal syndrome (CBS),[6] or primary progressive aphasia (PPA) semantic variant or nonfluent variant[7]; or United Kingdom Brain Bank criteria for idiopathic Parkinson's disease.[8] Participants enrolled in the CVD cohort had MRI- or CT-confirmed ischemic stroke at least three months prior to recruitment, with or without cognitive impairment. All participants were required to have a Montreal Cognitive Assessment (MoCA) score ≥18 (≥14 for participants with atypical Alzheimer's disease or FTD)[9]; note that the Parkinson's disease and CVD cohorts intentionally included participants both with and without cognitive impairment based on MoCA score.

Eye movement recording and task paradigm

Participants were seated in a dark room approximately 60cm away from a 17-inch computer monitor with 1280x1024 pixel resolution. Monocular eye position was tracked using an infrared video-based eye tracker (EyeLink 1000 Plus; SR Research Ltd., Ottawa, ON, Canada) at a sampling rate of 500 Hz. A nine-point array calibration was performed for each participant prior to task initiation whenever possible, with occasional use of a five-point array if the nine-point array proved unsuccessful.

All participants completed an interleaved pro- and anti-saccade task (IPAST)[10] consisting of two blocks of 120 trials (Supplementary Fig. 2). Following an intertrial interval lasting 1000ms, each trial began with the appearance of a central fixation point (0.5º diameter; 42 cd/m^2) lasting 1000ms and displayed on a black background (0.1 cd/m^2). The fixation point could be one of two luminance-matched colours that each indicated a different task instruction (green = prosaccade, red = antisaccade). The fixation point then disappeared for a gap period of 200ms, during which the screen remained empty, followed by the appearance of a peripheral stimulus (0.5º diameter; 42 cd/m^2) at 10º horizontally to either the left or right of the fixation position. On prosaccade trials, participants were instructed to make a saccade to the stimulus location as quickly as possible; on antisaccade trials, they were instructed not to look at the stimulus and instead to look in the opposite direction from where it appeared. Saccades in the opposite direction from the stimulus during prosaccade trials (which occur infrequently) and saccades towards the stimulus during antisaccade trials (which occur frequently) were considered direction errors.

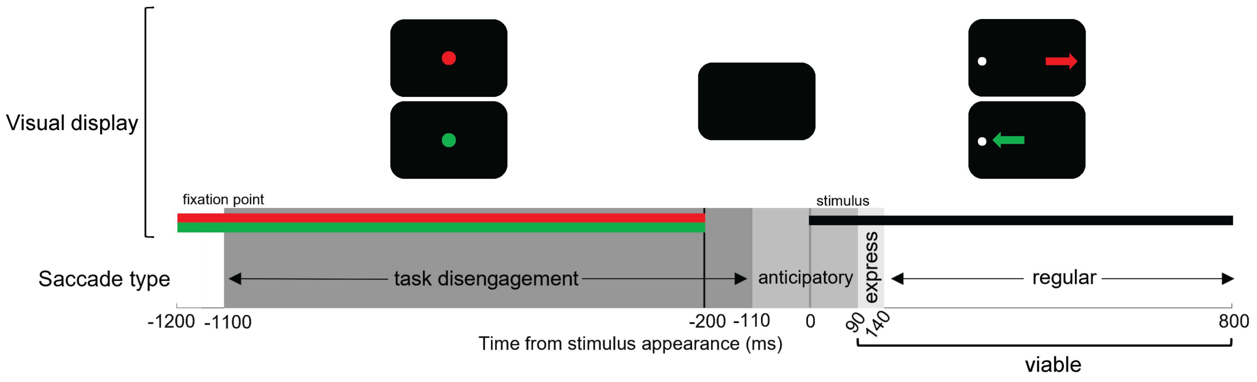

Supplementary Figure 2. Interleaved pro- and anti-saccade task (IPAST) visual display and saccade latency classification. X-axis shows time in milliseconds relative to peripheral stimulus appearance. Stimuli displayed on the screen are indicated in the top rows: fixation point (-1200 ms to -200 ms; green=prosaccade trial; red=antisaccade trial), gap period without visual stimulus (-200 ms to 0 ms), and peripheral stimulus (0 ms onward; green arrow indicates correct prosaccade, red arrow indicates correct antisaccade). Latency windows for saccade classification are indicated by grey boxes and corresponding labels in the bottom row: task disengagements (occur -1100 ms to -111 ms), anticipatory saccades (occur -110 ms to 89 ms), express latency saccades (occur 90 ms-139 ms), and regular latency saccades (occur 140 ms-800 ms). Viable saccades encompass both express and regular latency saccades.

Cognitive domain scores

As described elsewhere,[11,12] cognitive domain scores were calculated for all ONDRI participants using 23 test scores from the neuropsychology battery[13] for each of five cognitive domains: attention/working memory, executive function, language, memory, and visuospatial function. Standardized residuals were computed for each raw test score using a linear model with age, sex, and years of education as independent variables. Test version was also included where applicable (Symbol Digit Modality Test written vs oral version; Delis-Kaplan Executive Function System – Verbal Fluency standard vs alternate version). Timed scores were inverted so that higher scores signified better performance. Residuals for each test were grouped according to cognitive domain based on established neuropsychological convention[14,15] and consensus among ONDRI neuropsychologists (Supplementary Table 1); residuals were then averaged within each domain to produce a single score per participant per domain. Note that although most participants completed the Brief Visuospatial Memory Test – Revised (BVMT-R), including timed recall trials that can be used to measure non-verbal memory, it was not used in the calculation of memory scores as it was not completed by the ALS cohort. This ensured that memory scores were calculated identically across all cohorts; note therefore that the memory score represents verbal memory only.

Cognitive status

Cognitive status was used to subdivide ALS, Parkinson's disease, and CVD into one of four categories: cognitively normal, mild cognitive impairment (MCI), dementia, or other (individuals who did not clearly meet criteria for another category). This classification was based on three metrics: 1) cognitive performance on the neuropsychology test battery; 2) subjective cognitive

decline (self- or study partner-report) as assessed by the Short Informant Questionnaire on Cognitive Decline in the Elderly (Short IQ-CODE)[16]; and 3) activities of daily living (ADLs) assessed using an instrumental ADLs questionnaire adapted from Lawton *et al.*[17] and completed by participants' study partners.

Tests comprising the neuropsychology battery were divided into five cognitive domains (attention/working memory, executive function, language, memory, visuospatial function) as shown in Supplementary Table 1. Each individual test score was considered abnormal if the participant scored ≥1.5SD below age- and sex-corrected norms. In the ALS and CVD cohorts, the overall neuropsychology battery was considered impaired if the participant had at least 2 abnormal scores within a single domain. In the Parkinson's disease cohort, the requirement for multiple domains was dropped and the overall neuropsychology battery was considered impaired if the participant had at least 2 abnormal scores regardless of cognitive domain, in accordance with established criteria.[15]

Subjective cognitive decline was considered present if participants or their study partners reported cognitive decline (responding either "a bit worse" or "much worse") on at least one of the sixteen everyday cognitive tasks probed by Short IQ-CODE (e.g. "Compared to 10 years ago, how are you/the participant at recalling conversations a few days later?"). Subjective cognitive decline was not considered present if all responses reported no change or improvement.

ADLs were assessed using a questionnaire on which participants' study partners were asked to rate the participants' capacity for independent function in each of eight types of daily tasks (using the telephone, doing laundry, shopping, using transportation, preparing food, managing medication, home maintenance, and finances). ADLs were considered intact or minimally impacted if study partners provided only "A" or "B" responses (corresponding to full independence or limited support; e.g., "does personal laundry completely" and "launders small items, rinses socks, etc." respectively) with no "C", "D", or "E" responses (corresponding to increasing degrees of required support from others).

Participants were considered cognitively normal if they demonstrated normal cognition on the test battery and had intact ADLs, regardless of subjective cognitive decline. Participants who demonstrated cognitive impairment on the test battery and reported a subjective decline in cognition, but whose ADLs remained intact or minimally impacted, were classified as MCI; those who showed cognitive impairment on the test battery across multiple cognitive domains (abnormal scores in at least two different domains), endorsed subjective cognitive decline, and had impaired ADLs were considered to have dementia. Participants who did not meet criteria for any of these categories were classified as other. In the Parkinson's disease cohort, cognitive impairment was considered present on the neuropsychology battery if the participant scored ≥1.5SD below education- and/or age-corrected norms on at least two measures, according to the recommendations of the Movement Disorder Society Task Force for classifying MCI in Parkinson's disease.[15] In ALS and CVD, cognitive impairment was considered present if the

participant scored ≥1.5SD below norms on at least two measures within the same cognitive domain (Supplementary Table 1), as in previous work[18]. Additionally, impairment on any of multiple interrelated measures were considered only one impairment within a domain. Note that although the BVMT-R recall trials were not used in the calculation of memory domain scores, it was included where possible (i.e. in the Parkinson's disease and CVD cohorts) in evaluation of overall cognitive status.

Ultimately, this classification resulted in four subgroups within each of the ALS, Parkinson's disease, and CVD cohorts: cognitively normal (CN), MCI, dementia (D), and other. However, due to the small size of the ALS-MCI and ALS-D subgroups, we combined these into a single subgroup called ALS-CI (cognitively impaired). We note that this may have excluded some ALS participants who were classified as Other because they demonstrated impaired iADLs, which could have been due to disease-related physical rather than cognitive impairment, despite their normal cognitive performance on the neuropsychology battery.

Missing neuropsychology data was imputed with the worst possible score if missing because the participant was too impaired to complete the task (41 scores total across all tests and participants), or with the variable mean if missing for non-disease-related reasons (133 scores total across all tests and participants).

Although different classification methods were used to subdivide each cohort by cognitive status, we only performed statistical comparisons for each subgroup to the control cohort and to the other subgroups within the same cohort. No subgroups generated using different classification methods were directly compared.

Supplementary Table 1. Grouping of neuropsychology test measures into cognitive domains.

Cognitive domain	Attention & working memory	Executive function	Language	Verbal memory	Visuospatial function
**Neuropsychology test measures**	• Symbol digit modality test (coding)<br>• Trail making test – Part A (time)<br>• WAIS-III: digit span forward<br>• WAIS-III: digit span backward<br>• WAIS-III: digit span total<br>• DKEFS: colour naming (time)<br>• DKEFS: word reading (time)	• Trail making test – Part B (time)<br>• DKEFS: interference (time)<br>• DKEFS: switching (time)<br>• DKEFS: letter fluency<br>• DKEFS: category fluency<br>• WASI-II: matrix reasoning	• Boston naming – 15 item (pro-rated)<br>• TAWF: verb naming<br>• BDAE: semantic probe<br>• WASI-II: vocabulary	• RAVLT: immediate<br>• RAVLT: long-delay<br>• RAVLT: recognition discrimination	• Judgment of line orientation<br>• VOSP: incomplete letters<br>• BVMT-R: copy trial

WAIS-III: Wechsler Adult Intelligence Scale – Third Edition; DKEFS: Delis-Kaplan Executive Function System; WASI-II: Wechsler Abbreviated Scale of Intelligence, Second Edition; TAWF: Test of Adolescent/Adult Word Finding; BDAE: Boston Diagnostic Aphasia Examination; RAVLT: Rey Auditory Verbal Learning Task; BVMT-R: Brief Visuospatial Memory Test – Revised; VOSP: Visual Object and Space Perception Battery.

Saccade classification

Saccade data was preprocessed[19] and saccades were then categorized by an auto-marking script written in MATLAB (The MathWorks, Inc., Natick, MA, USA). We excluded trials with poor data quality or significant behavioural aberrations as described in the main text. Saccades were subsequently classified based on saccadic reaction time (SRT) (Supplementary Fig. 2) (time between peripheral stimulus appearance and saccade initiation).

A minimum of 90ms is required for a visual signal to propagate through the oculomotor system and trigger a saccade.[20] Therefore, any saccades occurring 90-800ms post-stimulus appearance were considered viable task-relevant responses made with perception of the stimulus location. Viable saccades were further subdivided based on latency and correctness. Saccades occurring 90ms to 139ms after stimulus appearance were classified as express latency saccades, and those occurring 140ms to 800ms after stimulus appearance were classified as regular latency saccades. Saccades made in a direction consistent with task instruction on a given trial were considered correct; those made in the opposite direction were considered direction errors. Only the first saccade initiated was considered for analysis; any subsequent corrections were not analyzed. Saccades with reaction time >800ms occur rarely and were therefore considered outliers and excluded from analysis.

Due to the abovementioned 90ms delay, the participant perceives the screen to be empty from 110ms before to 89ms after stimulus appearance, so any saccades initiated during this period were equally likely to be correct or incorrect and were considered indicative of guessing behaviour. These saccades were classified as anticipatory saccades.

Saccades were considered task disengagements if the participant looked away from the fixation point while they perceived it on the screen (1110ms before to 111ms before stimulus appearance).

Reaction times of initial prosaccades and antisaccades were also measured, as well as peak velocity (degrees/s), and amplitude (distance between the starting point of the initial saccade and its endpoint in degrees) for initial viable correct prosaccades only.

Saccade parameter calculation

We focused on 12 specific parameters that captured behaviour at different times across the tasks. Also see Supplementary Table 2.

To characterize behaviour early in the task, we identified trials in which participants looked away from the fixation point and never returned, as a measure of task disengagement. The percentage of prosaccade task disengagements was determined by calculating the percentage of prosaccade task disengagements out of all prosaccade trials completed excluding trials with technical issues (e.g., lost tracking, bad calibration). Percentage of antisaccade task disengagements was determined identically but using antisaccade trials only.

To quantify and characterize guessing behaviour occurring during the brief interval following fixation disappearance and prior to stimulus presentation, when the participant perceives a blank screen, we measured the percentage of anticipatory saccades. The percentage of anticipatory prosaccades was determined by calculating the percentage of anticipatory prosaccades out of all prosaccade trials completed excluding trials with technical issues; percentage of anticipatory antisaccades was determined identically using antisaccade trials instead.

Percentage of express latency correct prosaccades was determined by calculating the percentage of express latency correct prosaccades out of all *viable* prosaccades only. This parameter was chosen to understand participants' prosaccade behaviour during the early portion of the stimulus presentation epoch and because it depends on excitability levels in oculomotor circuits at the time of stimulus appearance.[21]

Percentages of express latency antisaccade errors and regular latency antisaccade errors were determined by calculating the percentage of express latency antisaccade errors and regular latency antisaccade errors respectively only out of all *viable* antisaccade trials. By separating these parameters, we were able to characterize error behaviours occurring at both short and longer latencies during the stimulus presentation epoch.

Mean prosaccade SRT and antisaccade SRT for each participant were determined based only on viable correct prosaccades or antisaccades respectively. Participants were also required to have at least five viable correct prosaccades or antisaccades for this measure to be calculated to ensure representative values; if they did not meet this criterion, the parameter was not computed for that participant. Mean prosaccade velocity and mean prosaccade amplitude were also calculated based only on viable correct prosaccades and required five viable correct prosaccades. We did not quantify the velocity and amplitude of antisaccades because there was considerable variability in antisaccade endpoints among participants, who were not instructed to make accurate antisaccades but only to look to the side opposite the stimulus. These parameters enabled us to characterize the overall speed of participants' task-appropriate prosaccade and antisaccade responses.

VOT was calculated by first generating a curve for each participant displaying the cumulative percentage of correct antisaccades and a second curve for percentage of antisaccade direction errors, then subtracting the error curve from the correct curve. This produces a characteristic curve whose nadir indicates the time during antisaccade trials after which voluntary processes begin to outcompete automated visually driven signals to produce correct antisaccades.[10,22] VOT was determined as the time in milliseconds after stimulus appearance at which this nadir occurred. It was restricted from 90-300ms following stimulus appearance and required at least 10 antisaccades at any latency. This parameter was selected to understand when during the trial participants' voluntary motor programs were able to outcompete automated ones.

Supplementary Table 2. Eye tracking measures of interest and calculation details.

Measure	Calculation	Additional requirements
**% Prosaccade task disengagements**	100*(number of prosaccade task disengagements/total prosaccade trials)	-
**% Antisaccade task disengagements**	100*(number of antisaccade task disengagements/total antisaccade trials)	-
**% Anticipatory prosaccades**	100*(number of anticipatory prosaccades/total prosaccade trials)	-
**% Anticipatory antisaccades**	100*(number of anticipatory antisaccades/total antisaccade trials)	-
**Mean prosaccade SRT**	Mean reaction time of all viable correct prosaccades for each participant	Participant must have ≥5 viable correct prosaccades
**Mean antisaccade SRT**	Mean reaction time of all viable correct antisaccades for each participant	Participant must have ≥5 viable correct antisaccades
**% Express latency correct prosaccades**	100*(number of express latency correct prosaccades/all viable prosaccade trials)	-
**% Express latency antisaccade direction errors**	100*(number of express latency antisaccade errors/all viable antisaccade trials)	-
**% Regular latency antisaccade direction errors**	100*(number of regular latency antisaccade errors/all viable antisaccade trials)	-
**VOT**	Subtract each participant's cumulative antisaccade error curve from correct curve; VOT is the time after stimulus appearance (ms) at the nadir of the resultant curve	Participant must have ≥10 antisaccades (any latency)
**Mean prosaccade velocity**	Mean peak velocity value of all viable correct prosaccades for each participant	Participant must have ≥5 viable correct prosaccades
**Mean prosaccade amplitude**	Mean amplitude of all viable correct prosaccades for each participant	Participant must have ≥5 viable correct prosaccades

SRT: saccadic reaction time; VOT: voluntary override time

Quality control and group analysis exclusion criteria

Of the 520 recruited ONDRI participants, 485 successfully completed eye tracking assessment (Supplementary Fig. 3A, B). Additional quality control criteria were then applied across all groups to remove participants with low-quality eye tracking data from all subsequent group and factor analyses. All participants were required to have completed at least 120 total IPAST trials (Supplementary Fig. 3C), of which at least 80% were required to have appropriate calibration and no loss of tracking (Supplementary Fig. 3D). A minimum of five viable prosaccades and five viable antisaccades were also required to mitigate the effects of potentially unrepresentative or noisy parameter values derived from a very small number of instances– for example, an antisaccade SRT value for a participant who made only one viable correct antisaccade. These criteria eliminated 35 ONDRI participants and no control participants, resulting in 450 ONDRI and 149 control participants (Supplementary Fig. 3E).

We included all ONDRI participants in the factor analysis, regardless of subtype or cognitive status, but required that they have no missing data. Practically, this excluded only participants who made too few viable correct antisaccades to accurately estimate their antisaccade SRT and resulted in 391 ONDRI participants included in the final factor analysis (Supplementary Fig. 3F). Control participants were excluded since they did not complete neuropsychological assessment and could not be included in subsequent analyses investigating relationships between factor scores and neuropsychology data. Demographic characteristics of participants included in factor analysis are displayed in Table 1 and Supplementary Fig. 1.

We removed very small and/or poorly defined subgroups (atypical Alzheimer's disease, progressive nonfluent aphasia, semantic dementia, corticobasal syndrome, participants with cognitive status "other", and one participant with both bvFTD and PSP) from parameter-level analysis. This resulted in 344 ONDRI and 149 control participants included.

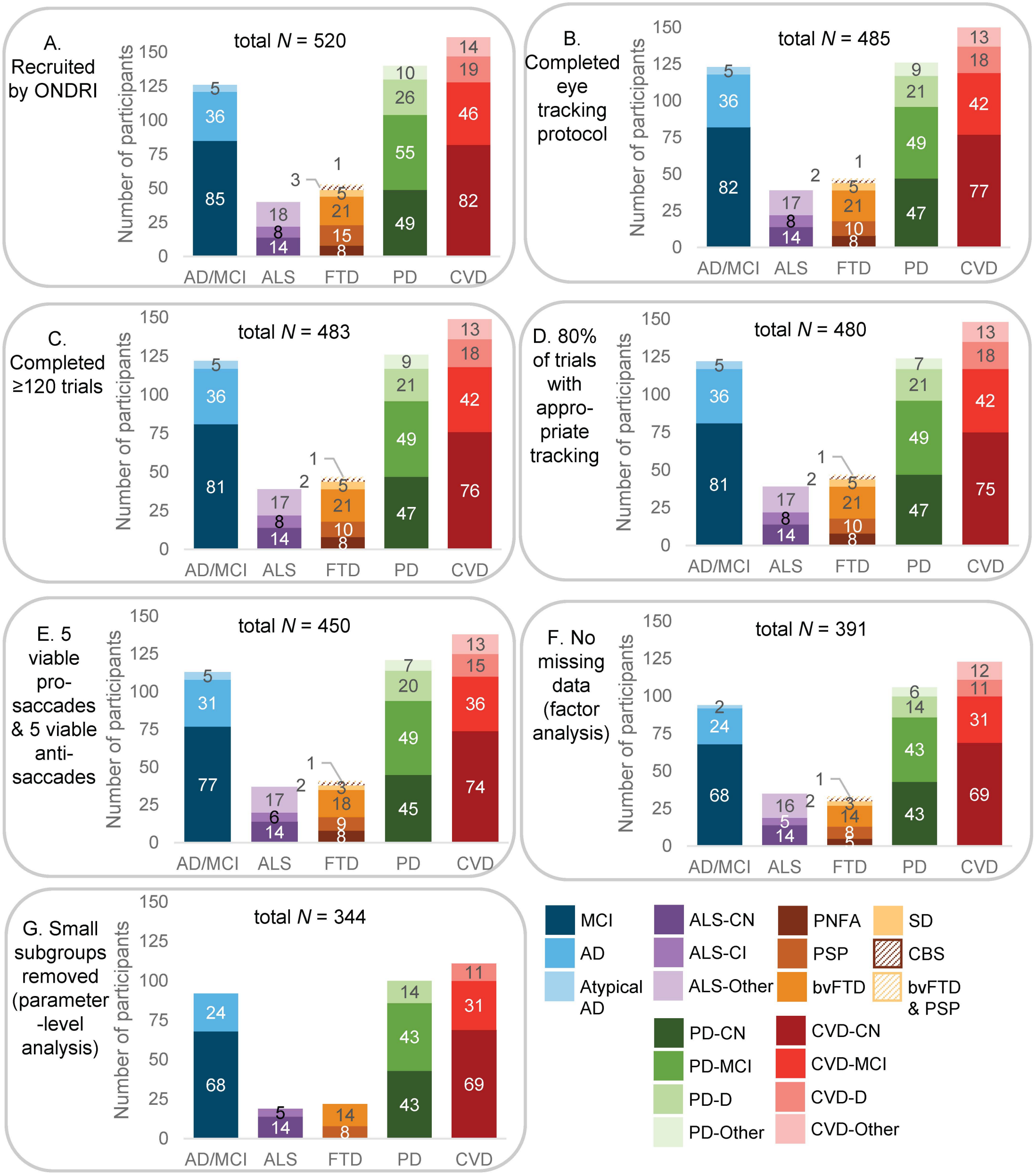

Supplementary Figure 3. Number of ONDRI participants by cohort and subgroup remaining after each exclusion criterion. (**A**) All participants initially recruited by ONDRI; (**B**) All participants who successfully completed eye tracking; (**C**) All participants with at least 120 completed trials; (**D**) All participants with at least 80% of trials having appropriate tracking (i.e. no tracking loss or inappropriate calibration); (**E**) All participants who made at least 5 viable prosaccades and 5 viable antisaccades; (**F**) All participants included in factor analysis; (**G**) All participants included in parameter-level analysis. MCI: mild cognitive impairment. AD: Alzheimer's disease. ALS: amyotrophic lateral sclerosis. bvFTD: behavioural variant frontotemporal dementia. PSP: progressive supranuclear palsy. CBS: corticobasal syndrome. PNFA: progressive nonfluent aphasia. SD: semantic dementia. PD: Parkinson's disease. CVD: cerebrovascular disease. CN: cognitively normal. CI: cognitively impaired. D: dementia. Other: cognitive status unclear.

Supplementary Results

Factor analysis structure matrix

Supplementary Table 3. Structure matrix for factor analysis.

Variable	Factor 1	Factor 2	Factor 3	Factor 4
**Prosaccade task disengagements**	**-0.85**	0.21	0.13	0.004
**Antisaccade task disengagements**	**-0.83**	0.13	0.15	0.05
**Prosaccade SRT**	0.14	**-0.75**	0.32	-0.21
**Anticipatory prosaccades**	-0.28	**0.59**	-0.16	0.19
**Anticipatory antisaccades**	-0.31	**0.52**	-0.09	0.16
**Express correct prosaccades**	-0.07	**0.93**	-0.23	0.04
**Express antisaccade errors**	-0.09	**0.78**	-0.04	-0.04
**Regular antisaccade errors**	-0.28	-0.03	**0.74**	-0.18
**Voluntary override time**	-0.05	-0.22	**0.96**	-0.20
**Antisaccade SRT**	0.08	-0.40	**0.67**	-0.39
**Prosaccade velocity**	-0.03	0.10	-0.16	**0.62**
**Prosaccade amplitude**	-0.02	0.03	-0.28	**0.98**

Note: *N*=391; extraction method: principal axis factoring; rotation method: oblique (direct oblimin with Kaiser normalization). Factor loadings above 0.3 are in bold.

Cumulative distributions of saccades

Cumulative distributions of saccadic reaction times were constructed from the raw data (i.e. uncorrected for age and sex) to summarize the behaviour of subgroups within each cohort (Supplementary Fig. 4) and qualitatively evaluated for differences between groups. Visual inspection indicated that subgroups largely did not differ in behaviour on prosaccade trials within or across cohorts. On antisaccade trials, however, more cognitively impaired subgroups displayed worse performance relative to less cognitively impaired subgroups, as indicated by consistently shallower slopes in the correct curve (i.e., slower correct antisaccade reaction time) and overall lower correct and higher error curves (i.e., higher proportion of antisaccade direction errors). Disease subgroups, particularly those with cognitive impairment, displayed generally worse performance than controls. We additionally computed cumulative distributions for the control data based on a median split by age (Supplementary Fig. 4F), which indicated that even the older group of controls demonstrated better antisaccade performance than patient groups.

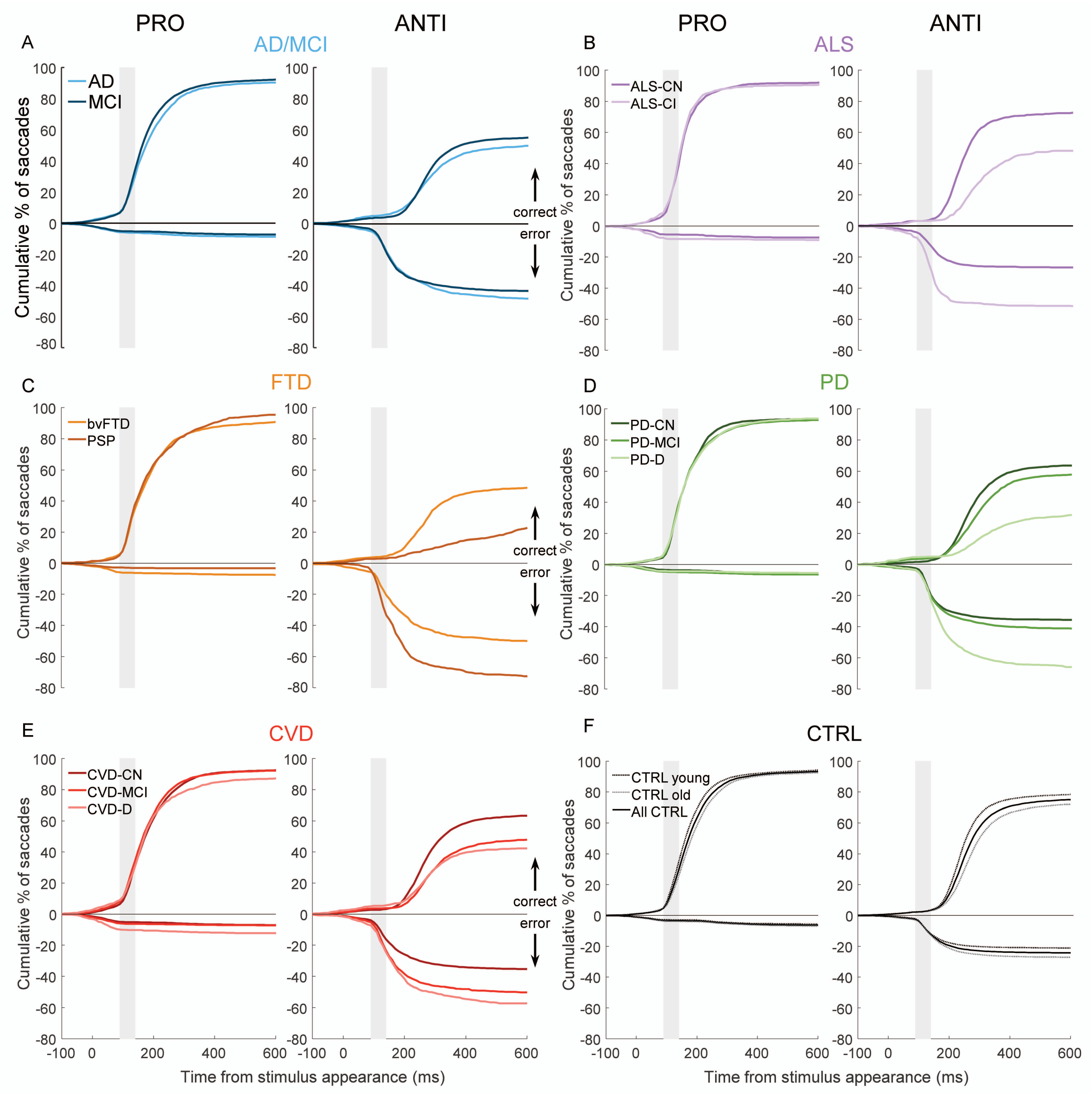

Supplementary Figure 4. Cumulative distributions of correct and error trials by latency across cohorts. (**A**) Alzheimer's disease (AD)/mild cognitive impairment (MCI) cohort divided into AD and MCI subgroups; (**B**) Amyotrophic lateral sclerosis (ALS) cohort divided into cognitively normal (CN) and cognitively impaired (CI) subgroups; (**C**) Frontotemporal dementia cohort divided into behavioural variant frontotemporal dementia (bvFTD) and progressive supranuclear palsy (PSP) subgroups; (**D**) Parkinson's disease (PD) cohort divided into cognitively normal (CN), mild cognitive impairment (MCI) and dementia (D) subgroups; (**E**) Cerebrovascular disease (CVD) cohort divided into cognitively normal (CN), mild cognitive impairment (MCI) and dementia (D) subgroups; (**F**) Control cohort, including median split by age into young (black dotted line) and old (grey dotted line) subgroups. Left panel in each pair indicates prosaccade (PRO) cumulative distribution; right panel indicates antisaccade (ANTI). X-axes indicate time relative to appearance of the peripheral stimulus. Grey bars indicate the express latency saccade window (90-139ms after stimulus appearance).

Additional parameter-level results

Anticipatory saccades occur at latencies when the participant perceives a blank screen and are equally likely to occur in either direction; they are therefore considered indicative of impulsive guessing behaviour. The percentage of anticipatory prosaccades was significantly different across groups ($H(12)=32.94$, $P<0.001$) with the MCI ($z=3.28$, $P=0.021$) and CVD-MCI ($z=3.53$, $P=0.0087$) groups making significantly more anticipatory prosaccades than controls (Supplementary Fig. 5A). A Kruskal-Wallis test indicated significant between-group differences in anticipatory antisaccades ($H(12)=24.68$, $P=0.016$); however, no post hoc tests were significant (Supplementary Fig. 5B).

There were no differences between groups in prosaccade reaction time ($H(12)=17.98$, $P=0.12$) (Supplementary Fig. 5C). There were, however, significant differences between groups in antisaccade reaction time ($H(12)=39.14$, $P<0.001$) (Supplementary Fig. 5D). Post hoc testing indicated that the PSP ($z=3.17$, $P=0.030$), PD-MCI ($z=3.00$, $P=0.048$), and PD-D ($z=3.54$, $P=0.0084$) groups displayed slower antisaccade reaction time than controls. PD-D participants also displayed slower antisaccade reaction time than PD-CN participants ($z=3.12$, $P=0.035$).

We tested for between-group differences in express latency correct prosaccades. Prosaccades at this latency typically reflect the integrity of visuomotor circuitry that generates a visually-driven saccade. There were no differences between groups in express latency correct prosaccades ($H(12)=17.92$, $P=0.12$) (Supplementary Fig. 5E).

There were no significant differences in prosaccade velocity ($H(12)=18.52$, $P=0.10$) (Supplementary Fig. 5F).

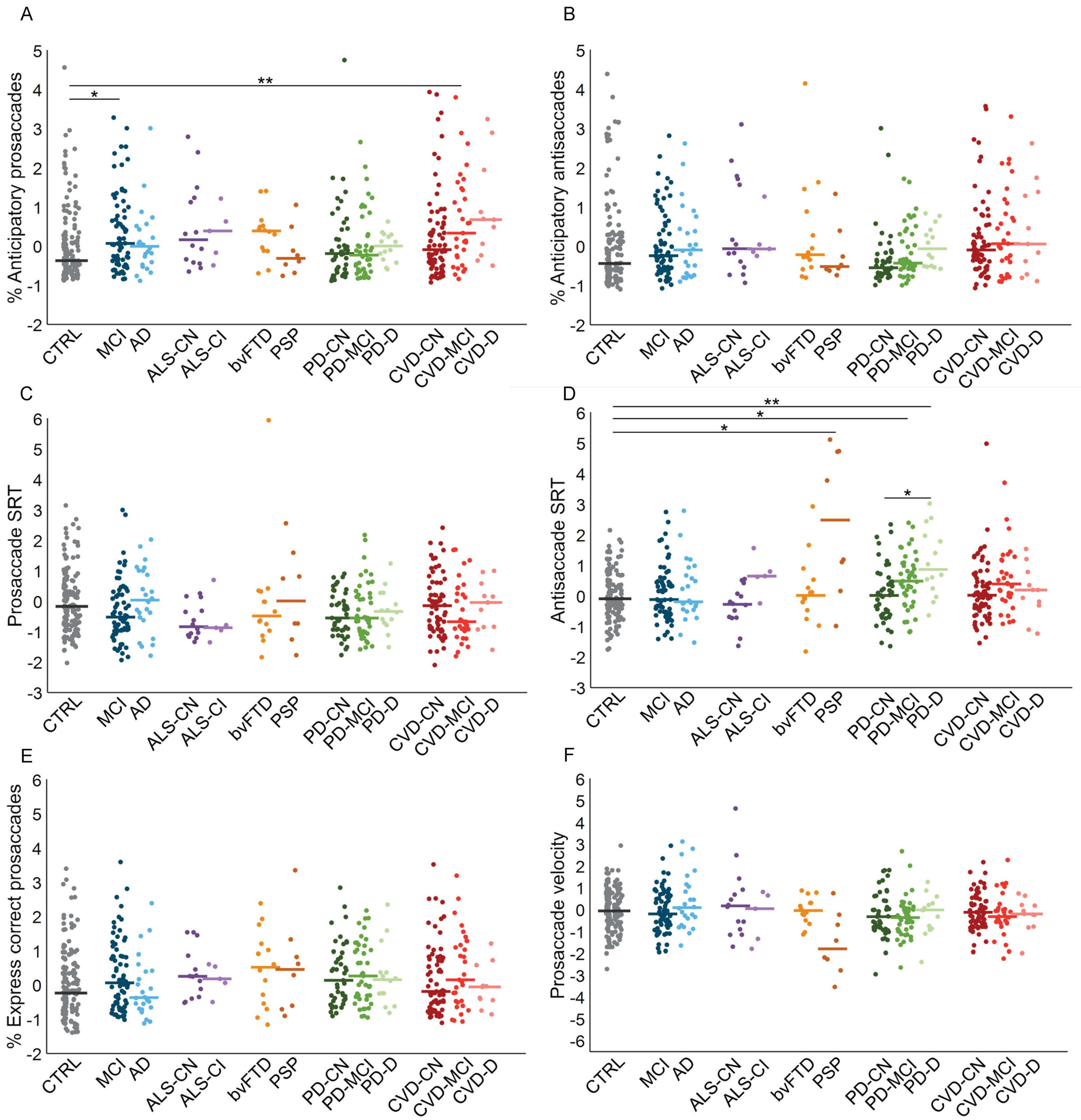

Supplementary Figure 5. Age- and sex-corrected eye tracking measures by subgroup. (**A**) Standardized residual of percentage of anticipatory prosaccades; (**B**) Standardized residual of percentage of anticipatory antisaccades; (**C**) Standardized residual of prosaccade reaction time; (**D**) Standardized residual of antisaccade reaction time; (**E**) Standardized residual of percentage of express latency correct prosaccades; (**F**) Standardized residual of prosaccade velocity. Each point represents a single participant; horizontal lines show subgroup medians. *p<0.05; **p<0.01; ***p<0.001 (Kruskal-Wallis tests followed by post hoc Dunn's tests with Holm-Bonferroni correction for multiple comparisons). CTRL: control; MCI: mild cognitive impairment; AD: Alzheimer's disease; ALS: amyotrophic lateral sclerosis; bvFTD: behavioural variant frontotemporal dementia; PSP: progressive supranuclear palsy; PD: Parkinson's disease; CVD: cerebrovascular disease; CN: cognitively normal; CI: cognitively impaired; D: dementia.

Supplementary Discussion

Disease-specific findings

Many existing studies have examined various antisaccade parameters in a variety of neurodegenerative and cerebrovascular conditions. We situate the results from this study in context of prior disease-specific analyses.

Alzheimer's disease and mild cognitive impairment

Most studies have focused on Alzheimer's disease alone, with a minority also including an MCI comparison group and a smaller minority examining MCI only. The most reported finding is increased error rates in disease groups relative to controls, which we replicated in Alzheimer's disease[23-37] and MCI.[32,33,35,38] However, we did not corroborate the finding in some studies that Alzheimer's disease participants generated more errors than MCI participants[39,40] or that MCI participants did not differ from controls.[39,41] Together, these results suggest that antisaccade error rate robustly detects Alzheimer's disease/MCI but does not differentiate between prodromal and full-blown states; clinical overlap between and heterogeneity within these subgroups may complicate efforts to develop behavioural biomarkers that separate them by appraising underlying neural dysfunction. Some recent work involving both amnestic and nonamnestic MCI participants indicates that amnestic MCI participants have similar error rates to Alzheimer's disease patients and make more errors than nonamnestic MCI participants[36,42]; in line with this, our study included only amnestic MCI and found no differences between them and Alzheimer's disease participants. Future work should further probe this distinction.

Saccadic reaction times are also frequently reported, but findings are mixed. Alzheimer's disease and/or MCI have been described as having either slower prosaccade reaction times than controls,[24,28,29,37,41-48] or no difference,[25-27,32,38,40,49,50] with similarly inconclusive findings in antisaccade reaction time. Our results suggest that findings of slowed saccadic reaction time may not hold over many trials (120 of each type as opposed to many fewer trials in most studies).

Our results do not clearly validate the small number of previous reports of increased anticipatory saccades in Alzheimer's disease[24,32,33] but we did find increased anticipatory prosaccades in MCI. We also corroborate the majority of findings indicating no differences from controls in saccade amplitude or velocity,[25-29,31,44-47] with a few exceptions.[24,33,49]

Amyotrophic lateral sclerosis

Neither cognitively normal nor cognitively impaired ALS participants in our study displayed any differences from controls. This is particularly in opposition to previous studies of ALS that have typically found increased antisaccade errors in ALS. However, studies with larger sample sizes have more consistently found higher error rates in ALS[51-54] than those with small sample sizes.[55,56] The lack of significance in this study may therefore be due to the comparatively

small size of our ALS subgroups. We also did not replicate a few previous findings of slowed antisaccade reaction time,[51-53] although this is not universal.[57] Clinical heterogeneity may also play a role but was considered outside the scope of this study.

Frontotemporal dementia

We did not divide the FTD cohort into cognition-based subgroups, but observed results in line with previous work finding increased antisaccade errors relative to controls in PSP.[28,29,58-61] Our results regarding saccadic reaction time in PSP were in line with studies indicating slowed antisaccade rection time[60,61] although some studies found no difference[28,62]; and no difference in prosaccade reaction time[28,29,58-62] with one exception.[63] We also replicated a very consistent finding of reduced saccade amplitude, but our results did not support reports of reduced saccade velocity,[28,29,59,62,64,65] although this may relate to statistical power issues as the median saccade velocity appeared much lower in PSP.

We did not observe an increase in errors in the bvFTD subgroup, in opposition to existing literature indicating more errors in bvFTD alone or a pooled FTD group relative to controls.[27-29,60,66] We found no differences between bvFTD and controls in prosaccade and antisaccade reaction time, saccade amplitude, or saccade velocity.

Parkinson's disease

Many studies have described antisaccade behaviour in Parkinson's disease. Most of these find increased error rates in Parkinson's disease compared to controls[61,67-78] which has also been confirmed by meta-analysis.[79] However, our finding that only PD-D had increased regular latency errors relative to controls, PD-CN, and PD-MCI indicates this relationship may be related to cognitive impairment, similar to Mosimann *et al.*[26] Medication effects[80,81] and disease severity[82] may also modulate error rates, which we did not control for, although note that participants classified as PD-D are likely to have longer disease course and may therefore have more severe disease.

Antisaccade reaction time is sometimes reported as being slowed in Parkinson's disease. A recent meta-analysis indicated increased antisaccade reaction time relative to controls,[79] which our results support. We also corroborate suggestions that cognitive impairment may modulate this relationship.[26] However, we did not find any slowing of prosaccade reaction time in Parkinson's disease, nor did we replicate findings that cognitive impairment may slow this metric further.[26,83]

Studies reporting express latency prosaccades typically find increases in Parkinson's disease,[69,77,84] which we did not replicate. However, there are some indications that dopaminergic medication may slow prosaccades[80,85]; note that ONDRI Parkinson's disease participants were all medicated. Finally, our study is in line with previous work showing that saccade amplitude is reduced in Parkinson's disease.

Cerebrovascular disease

Although only CVD-MCI demonstrated significantly more regular latency antisaccade errors than controls, there was a general qualitative trend towards increased errors with increased cognitive impairment. This result is novel due to the relative dearth of cohort studies of antisaccades in CVD. Existing cohort studies[86,87] and studies of localized ischemic lesions[88,89] collectively suggest that stroke location and size may be paramount in determining pro- and antisaccade behaviour in this group.

Voluntary override time and overall cognitive impairment

We additionally report the novel parameter VOT, which denotes the time taken for voluntary processes to begin overcoming automated processes during antisaccades.[10] As with regular latency antisaccade errors, cognitively impaired subgroups generally demonstrated poorer performance. In general, therefore, cognitive impairment probably slows initiation of voluntary processes and generates inhibitory control deficits.

Limitations & future directions

Due to the comprehensive nature of the study, we assessed several cohorts and subgroups of varying size, unequal variance, and clinical heterogeneity across numerous behavioural parameters. To offset the large number of between-group comparisons required by this design and limit type 1 errors, we employed Kruskal-Wallis tests, which offer less statistical power than typical parametric ANOVAs on normally distributed variables, and post hoc Dunn tests with a relatively conservative Holm-Bonferroni correction. Additionally, some cohorts and component subgroups (e.g. ALS, FTD) contained few participants. Therefore, the possibility of type II errors is nontrivial. Our findings, particularly those on infrequently reported IPAST parameters, should therefore be validated by cohort- or subgroup-specific studies in future.

Secondly, we did not attempt to control for clinical variables such as disease severity, medication type and dosage, or other disease-related parameters. However, most patients were taking medication and there is evidence that drugs used to manage neurodegenerative disease symptoms, especially dopaminergic anti-Parkinsonian medications, may affect oculomotor behaviour.[80,85] Exploration of the oculomotor effects of medication type, dosage, and other pertinent clinical parameters should be a target of future study.

This study is among a very small number to report pro- and antisaccade behaviour in CVD or related diseases. Behaviour in this cohort is difficult to typify at the group level; as demonstrated by lesion studies, stroke location determines the resultant behavioural deficits. We considered examination of IPAST behaviour according to stroke location to be beyond the scope of this study. Accordingly, the CVD cohort is best considered an illustration of our findings about cognitive impairment rather than as a uniform disease cohort. Since we did not exclude those with infarcts

in visual or oculomotor pathways, it is also possible that some members of this cohort performed poorly due to insufficient visual acuity or oculomotor ability. However, CVD collectively did not display differences from controls on prosaccade measures, indicating that visuomotor circuitry was intact for most participants. Stroke location should be considered in future investigation of this cohort.

Finally, although only baseline (first visit) data was reported in the current study, ONDRI participants completed the same assessments at follow-up visits for up to three years. Evaluation of cognitive decline over the time course of the study, and its relationship to IPAST parameters, should be completed as a supplement to the results described here and to understand how oculomotor behaviour may change with disease progression.

Supplementary References

1. McKhann GM, Knopman DS, Chertkow H, et al. The diagnosis of dementia due to Alzheimer's disease: Recommendations from the National Institute on Aging-Alzheimer's Association workgroups on diagnostic guidelines for Alzheimer's disease. *Alzheimer's and Dementia*. 2011;7(3):263-269. doi:10.1016/j.jalz.2011.03.005

2. Albert MS, DeKosky ST, Dickson D, et al. The diagnosis of mild cognitive impairment due to Alzheimer's disease: Recommendations from the National Institute on Aging-Alzheimer's Association workgroups on diagnostic guidelines for Alzheimer's disease. *Alzheimer's and Dementia*. 2011;7(3):270-279. doi:10.1016/j.jalz.2011.03.008

3. Brooks BR. El Escorial World Federation of Neurology criteria for the diagnosis of amyotrophic lateral sclerosis. *Journal of the Neurological Sciences*. 1994;124(SUPPL.):96-107. doi:10.1016/0022-510X(94)90191-0

4. Rascovsky K, Hodges JR, Knopman D, et al. Sensitivity of revised diagnostic criteria for the behavioural variant of frontotemporal dementia. *Brain*. 2011;134(9):2456-2477. doi:10.1093/brain/awr179

5. Litvan I, Agid Y, Calne D, et al. Clinical research criteria for the diagnosis of progressive supranuclear palsy (Steele-Richardson-Olszewski syndrome): Report of the NINDS-SPSP International Workshop. *Neurology*. 1996;47:1-9. doi:10.1212/wnl.47.1.1

6. Armstrong MJ, Litvan I, Lang AE, et al. Criteria for the diagnosis of corticobasal degeneration. *Neurology*. 2013;80(5):496-503. doi:10.1212/WNL.0b013e31827f0fd1

7. Gorno-Tempini ML, Hillis AE, Weintraub S, et al. Classification of primary progressive aphasia and its variants. *Neurology*. 2011;76(11):1006-1014. doi:10.1212/WNL.0b013e31821103e6

8. Hughes AJ, Daniel SE, Kilford L, Lees AJ. Accuracy of clinical diagnosis of idiopathic Parkinson's disease: A clinico-pathological study of 100 cases. *Journal of Neurology Neurosurgery and Psychiatry*. 1992;55(3):181-184. doi:10.1136/jnnp.55.3.181

9. Nasreddine ZS, Phillips NA, Bedirian V, et al. The Montreal Cognitive Assessment, MoCA: A Brief Screening Tool For Mild Cognitive Impairment. *J Am Geriatr Soc*. 2005;53(4):695-699. doi:10.1111/j.1532-5415.2005.53221.x

10. Yep R, Smorenburg ML, Riek HC, et al. Interleaved pro/anti-saccade behavior across the lifespan. *Frontiers in Aging Neuroscience*. 2022;14:842549. doi:10.3389/fnagi.2022.84254

11. Dilliott AA, Sunderland KM, McLaughlin PM, et al. Association of apolipoprotein E variation with cognitive impairment across multiple neurodegenerative diagnoses. *Neurobiology of Aging*. 2021;105:378.e1-378.e9. doi:10.1016/j.neurobiolaging.2021.04.011

12. Fishman KN, Roberts AC, Orange JB, et al. Bilingualism in Parkinson's disease: Relationship to cognition and quality of life. *Journal of Clinical and Experimental Neuropsychology*. 2021;43(2):199-212. doi:10.1080/13803395.2021.1902946

13. McLaughlin PM, Sunderland KM, Beaton D, et al. The Quality Assurance and Quality Control Protocol for Neuropsychological Data Collection and Curation in the Ontario Neurodegenerative Disease Research Initiative (ONDRI) Study. *Assessment*. 2021;28(5):1267-1286. doi:10.1177/1073191120913933

14. Lezak MD, Howieson DB, Loring DW, Hannay HJ, Fischer JS. Neuropsychological assessment. Published online 2004. doi:10.1037/10361-015

15. Litvan I, Goldman JG, Tröster AI, et al. Diagnostic criteria for mild cognitive impairment in Parkinson's disease: Movement Disorder Society Task Force guidelines. *Movement Disorders*. 2012;27(3):349-356. doi:10.1002/mds.24893

16. Jorm AF. A Short Form of the Informant Questionnaire on Cognitive Decline in the Elderly (IQCODE): Development and Cross-Validation. *Psychological Medicine*. 1994;24(1):145-153. doi:10.1017/S003329170002691X

17. Lawton MP, Brody EM. Assessment of older people: Self-maintaining and instrumental activities of daily living. *Gerontologist*. 1969;9(3):179-186. doi:10.1093/geront/9.3_Part_1.179

18. Zaidi KB, Rich JB, Sunderland KM, et al. Methods for improving screening for vascular cognitive impairment using the montreal cognitive assessment. *Canadian Journal of Neurological Sciences*. 2020;47(6):756-763. doi:10.1017/cjn.2020.121

19. Coe BC, Huang J, Brien DC, White BJ, Yep R, Munoz DP. Automated Analysis Pipeline For Extracting Saccade, Pupil, and Blink Parameters Using Video-Based Eye Tracking. *bioRxiv*. 2022;(6005163):1-29. doi:10.1101/2022.02.22.481518

20. Coe BC, Munoz DP. Mechanisms of saccade suppression revealed in the anti-saccade task. *Philosophical Transactions of the Royal Society B: Biological Sciences*. 2017;372:20160192. doi:10.1098/rstb.2016.0192

21. Dorris MC, Pare M, Munoz DP. Neuronal activity in monkey superior colliculus related to the initiation of saccadic eye movements. *Journal of Neuroscience*. 1997;17(21):8566-8579. doi:10.1523/JNEUROSCI.17-21-08566.1997

22. Coe BC, Trappenberg T, Munoz DP. Modeling saccadic action selection: Cortical and basal ganglia signals coalesce in the superior colliculus. *Frontiers in Systems Neuroscience*. 2019;13(3). doi:10.3389/fnsys.2019.00003

23. Abel LA, Unverzagt F, Yee RD. Effects of stimulus predictability and interstimulus gap on saccades in Alzheimer's disease. *Dementia and Geriatric Cognitive Disorders*. 2002;13(4):235-243. doi:10.1159/000057702

24. Shafiq-Antonacci R, Maruff P, Masters C, Currie J. Spectrum of saccade system function in Alzheimer disease. *Archives of Neurology*. 2003;60(9):1272-1278. doi:10.1001/archneur.60.9.1272

25. Crawford TJ, Higham S, Renvoize T, et al. Inhibitory control of saccadic eye movements and cognitive impairment in Alzheimer's disease. *Biological Psychiatry*. 2005;57(9):1052-1060. doi:10.1016/j.biopsych.2005.01.017

26. Mosimann UP, Müri RM, Burn DJ, Felblinger J, O'Brien JT, McKeith IG. Saccadic eye movement changes in Parkinson's disease dementia and dementia with Lewy bodies. *Brain*. 2005;128(6):1267-1276. doi:10.1093/brain/awh484

27. Boxer AL, Garbutt S, Rankin KP, et al. Medial versus lateral frontal lobe contributions to voluntary saccade control as revealed by the study of patients with frontal lobe degeneration. *Journal of Neuroscience*. 2006;26(23):6354-6363. doi:10.1523/JNEUROSCI.0549-06.2006

28. Garbutt S, Matlin A, Hellmuth J, et al. Oculomotor function in frontotemporal lobar degeneration, related disorders and Alzheimer's disease. *Brain*. 2008;131(5):1268-1281. doi:10.1093/brain/awn047

29. Boxer AL, Garbutt S, Seeley WW, et al. Saccade abnormalities in autopsy-confirmed frontotemporal lobar degeneration and alzheimer disease. *Archives of Neurology*. 2012;69(4):509-517. doi:10.1001/archneurol.2011.1021

30. Kaufman LD, Pratt J, Levine B, Black SE. Executive deficits detected in mild Alzheimer's disease using the antisaccade task. *Brain and Behavior*. 2012;2(1):15-21. doi:10.1002/brb3.28

31. Crawford TJ, Higham S, Mayes J, Dale M, Shaunak S, Lekwuwa G. The role of working memory and attentional disengagement on inhibitory control: Effects of aging and

Alzheimer's disease. *Age (Omaha)*. 2013;35(5):1637-1650. doi:10.1007/s11357-012-9466-y

32. Peltsch A, Hemraj A, Garcia A, Munoz DP. Saccade deficits in amnestic mild cognitive impairment resemble mild Alzheimer's disease. *European Journal of Neuroscience*. 2014;39(11):2000-2013. doi:10.1111/ejn.12617

33. Holden JG, Cosnard A, Laurens B, et al. Prodromal Alzheimer's Disease Demonstrates Increased Errors at a Simple and Automated Anti-Saccade Task. *Journal of Alzheimer's Disease*. 2018;65(4):1209-1223. doi:10.3233/JAD-180082

34. Noiret N, Carvalho N, Laurent É, et al. Saccadic eye movements and attentional control in Alzheimer's disease. *Archives of Clinical Neuropsychology*. 2018;33(1):1-13. doi:10.1093/arclin/acx044

35. Crawford TJ, Taylor S, Mardanbegi D, et al. The Effects of Previous Error and Success in Alzheimer's Disease and Mild Cognitive Impairment. *Scientific Reports*. 2019;9(1):20204. doi:10.1038/s41598-019-56625-2

36. Wilcockson TDW, Mardanbegi D, Xia B, et al. Abnormalities of saccadic eye movements in dementia due to Alzheimer's disease and mild cognitive impairment. *Aging*. 2019;11(15):5389-5398. doi:10.18632/aging.102118

37. Lage C, López-García S, Bejanin A, et al. Distinctive Oculomotor Behaviors in Alzheimer's Disease and Frontotemporal Dementia. *Frontiers in Aging Neuroscience*. 2021;12:603790. doi:10.3389/fnagi.2020.603790

38. Alichniewicz KK, Brunner F, Klünemann HH, Greenlee MW. Neural correlates of saccadic inhibition in healthy elderly and patients with amnestic mild cognitive impairment. *Frontiers in Psychology*. 2013;4(JUL). doi:10.3389/fpsyg.2013.00467

39. Hellmuth J, Mirsky J, Heuer HW, et al. Multicenter validation of a bedside antisaccade task as a measure of executive function. *Neurology*. 2012;78(23):1824-1831. doi:10.1212/WNL.0b013e318258f785

40. Chehrehnegar N, Shati M, Esmaeili M, Foroughan M. Executive function deficits in mild cognitive impairment: evidence from saccade tasks. *Aging and Mental Health*. 2022;26(5):1001-1009. doi:10.1080/13607863.2021.1913471

41. Heuer HW, Mirsky JB, Kong EL, et al. Antisaccade task reflects cortical involvement in mild cognitive impairment. *Neurology*. 2013;81(14):1235-1243. doi:10.1212/WNL.0b013e3182a6cbfe

42. Koçoğlu K, Hodgson TL, Eraslan Boz H, Akdal G. Deficits in saccadic eye movements differ between subtypes of patients with mild cognitive impairment. *Journal of Clinical and Experimental Neuropsychology*. 2021;43(2):187-198. doi:10.1080/13803395.2021.1900077

43. Hershey LA, Whicker L, Abel LA, Dell'osso LF, Traccis S, Grossniklaus D. Saccadic Latency Measurements in Dementia. *Archives of Neurology*. 1983;40(9):592-593. doi:10.1001/archneur.1983.04050080092023

44. Bylsma FW, Rasmusson DX, Rebok GW, Keyl PM, Tune L, Brandt J. Changes in visual fixation and saccadic eye movements in Alzheimer's disease. *International Journal of Psychophysiology*. 1995;19(1):33-40. doi:10.1016/0167-8760(94)00060-R

45. Yang Q, Wang T, Su N, Liu Y, Xiao S, Kapoula Z. Long Latency and High Variability in Accuracy-Speed of Prosaccades in Alzheimer's Disease at Mild to Moderate Stage. *Dementia and Geriatric Cognitive Disorders Extra*. 2011;1(1):318-329. doi:10.1159/000333080

46. Yang Q, Wang T, Su N, Xiao S, Kapoula Z. Specific saccade deficits in patients with Alzheimer's disease at mild to moderate stage and in patients with amnestic mild cognitive impairment. *Age (Omaha)*. 2013;35(4):1287-1298. doi:10.1007/s11357-012-9420-z

47. Crawford TJ, Devereaux A, Higham S, Kelly C. The disengagement of visual attention in Alzheimer's disease: A longitudinal eye-tracking study. *Frontiers in Aging Neuroscience*. 2015;7(JUN):1-10. doi:10.3389/fnagi.2015.00118

48. Polden M, Wilcockson TDW, Crawford TJ. The disengagement of visual attention: An eye-tracking study of cognitive impairment, ethnicity and age. *Brain Sciences*. 2020;10(7):1-13. doi:10.3390/brainsci10070461

49. Shakespeare TJ, Kaski D, Yong KXX, et al. Abnormalities of fixation, saccade and pursuit in posterior cortical atrophy. *Brain*. 2015;138(7):1976-1991. doi:10.1093/brain/awv103

50. de Boer C, van der Steen J, Mattace-Raso F, Boon AJW, Pel JJM. The effect of neurodegeneration on visuomotor behavior in Alzheimer's disease and Parkinson's disease. *Motor Control*. 2016;20(1):1-20. doi:10.1123/mc.2014-0015

51. Donaghy C, Pinnock R, Abrahams S, et al. Slow saccades in bulbar-onset motor neurone disease. *Journal of Neurology*. 2010;257(7):1134-1140. doi:10.1007/s00415-010-5478-7

52. Proudfoot M, Menke RAL, Sharma R, et al. Eye-tracking in amyotrophic lateral sclerosis: A longitudinal study of saccadic and cognitive tasks. *Amyotrophic Lateral Sclerosis and Frontotemporal Degeneration*. 2016;17(1-2):101-111. doi:10.3109/21678421.2015.1054292

53. Shaunak S, Orrell RW, O'Sullivan E, et al. Oculomotor function in amyotrophic lateral sclerosis: Evidence for frontal impairment. *Annals of Neurology*. 1995;38(1):38-44. doi:10.1002/ana.410380109

54. Yunusova Y, Ansari J, Ramirez J, et al. Frontal Anatomical Correlates of Cognitive and Speech Motor Deficits in Amyotrophic Lateral Sclerosis. *Behavioural Neurology*. 2019;2019. doi:10.1155/2019/9518309

55. Burrell JR, Carpenter RHS, Hodges JR, Kiernan MC. Early saccades in amyotrophic lateral sclerosis. *Amyotrophic Lateral Sclerosis and Frontotemporal Degeneration*. 2013;14(4):294-301. doi:10.3109/21678421.2013.783077

56. Witiuk K, Fernandez-Ruiz J, McKee R, et al. Cognitive Deterioration and Functional Compensation in ALS Measured with fMRI Using an Inhibitory Task. *Journal of Neuroscience*. 2014;34(43):14260-14271. doi:10.1523/JNEUROSCI.1111-14.2014

57. Evdokimidis I, Constantinidis TS, Gourtzelidis P, et al. Frontal lobe dysfunction in amyotrophic lateral sclerosis. *Journal of the Neurological Sciences*. 2002;195(1):25-33. doi:10.1016/S0022-510X(01)00683-9

58. Vidailhet M, Rivaud S, Gouider-Khouja N, et al. Eye movements in parkinsonian syndromes. *Annals of Neurology*. 1994;35(4):420-426. doi:10.1002/ana.410350408

59. Rivaud-Péchoux S, Vidailhet M, Gallouedec G, Litvan I, Gaymard B, Pierrot-Deseilligny C. Longitudinal ocular motor study in corticobasal degeneration and progressive supranuclear palsy. *Neurology*. 2000;54(5):1029-1032. doi:10.1212/WNL.54.5.1029

60. Meyniel C, Rivaud-Péchoux S, Damier P, Gaymard B. Saccade impairments in patients with fronto-temporal dementia. *Journal of Neurology, Neurosurgery and Psychiatry*. 2005;76(11):1581-1584. doi:10.1136/jnnp.2004.060392

61. Rivaud-Péchoux S, Vidailhet M, Brandel JP, Gaymard B. Mixing pro- and antisaccades in patients with parkinsonian syndromes. *Brain*. 2007;130(1):256-264. doi:10.1093/brain/awl315

62. Lemos J, Pereira D, Almendra L, et al. Cortical control of vertical and horizontal saccades in progressive supranuclear palsy: An exploratory fMRI study. *Journal of the Neurological Sciences*. 2017;373:157-166. doi:10.1016/j.jns.2016.12.049

63. Terao Y, Fukuda H, Shirota Y, et al. Deterioration of horizontal saccades in progressive supranuclear palsy. *Clinical Neurophysiology*. 2013;124(2):354-363. doi:10.1016/j.clinph.2012.07.008

64. Pinkhardt EH, Jürgens R, Becker W, Valdarno F, Ludolph AC, Kassubek J. Differential diagnostic value of eye movement recording in PSP-parkinsonism, Richardson's syndrome, and idiopathic Parkinson's disease. *Journal of Neurology*. 2008;255(12):1916-1925. doi:10.1007/s00415-009-0027-y

65. Terao Y, Tokushige S ichi, Inomata-Terada S, Fukuda H, Yugeta A, Ugawa Y. Deciphering the saccade velocity profile of progressive supranuclear palsy: A sign of latent cerebellar/brainstem dysfunction? *Clinical Neurophysiology*. Published online February 3, 2021. doi:10.1016/j.clinph.2020.12.023

66. Burrell JR, Hornberger M, Carpenter RHS, Kiernan MC, Hodges JR. Saccadic abnormalities in frontotemporal dementia. *Neurology*. 2012;78(23):1816-1823. doi:10.1212/WNL.0b013e318258f75c

67. Crevits L, de Ridder K. Disturbed striatoprefrontal mediated visual behaviour in moderate to severe parkinsonian patients. *Journal of Neurology Neurosurgery and Psychiatry*. 1997;63(3):296-299. doi:10.1136/jnnp.63.3.296

68. Briand KA, Strallow D, Hening W, Poizner H, Sereno AB. Control of voluntary and reflexive saccades in Parkinson's disease. *Experimental Brain Research*. 1999;129:38-48.

69. Chan F, Armstrong IT, Pari G, Riopelle RJ, Munoz DP. Deficits in saccadic eye-movement control in Parkinson's disease. *Neuropsychologia*. 2005;43(5):784-796. doi:10.1016/j.neuropsychologia.2004.06.026

70. Amador SC, Hood AJ, Schiess MC, Izor R, Sereno AB. Dissociating cognitive deficits involved in voluntary eye movement dysfunctions in Parkinson's disease patients. *Neuropsychologia*. 2006;44(8):1475-1482. doi:10.1016/j.neuropsychologia.2005.11.015

71. Blekher T, Weaver M, Rupp J, et al. Multiple step pattern as a biomarker in Parkinson disease. *Parkinsonism and Related Disorders*. 2009;15(7):506-510. doi:10.1016/j.parkreldis.2009.01.002

72. van Koningsbruggen MG, Pender T, Machado L, Rafal RD. Impaired control of the oculomotor reflexes in Parkinson's disease. *Neuropsychologia*. 2009;47(13):2909-2915. doi:10.1016/j.neuropsychologia.2009.06.018

73. Antoniades CA, Demeyere N, Kennard C, Humphreys GW, Hu MT. Antisaccades and executive dysfunction in early drug-naive Parkinson's disease: The discovery study. *Movement Disorders*. 2015;30(6):843-847. doi:10.1002/mds.26134

74. Gorges M, Müller HP, Lulé D, et al. The association between alterations of eye movement control and cerebral intrinsic functional connectivity in Parkinson's disease. *Brain Imaging and Behavior*. 2016;10(1):79-91. doi:10.1007/s11682-015-9367-7

75. Wang CA, McInnis H, Brien DC, Pari G, Munoz DP. Disruption of pupil size modulation correlates with voluntary motor preparation deficits in Parkinson's disease. *Neuropsychologia*. 2016;80:176-184. doi:10.1016/j.neuropsychologia.2015.11.019

76. Lu Z, Buchanan T, Kennard C, FitzGerald JJ, Antoniades CA. The effect of levodopa on saccades – Oxford Quantification in Parkinsonism study. *Parkinsonism and Related Disorders*. 2019;68(September):49-56. doi:10.1016/j.parkreldis.2019.09.029

77. Perkins JE, Janzen A, Bernhard FP, et al. Saccade, Pupil, and Blink Responses in Rapid Eye Movement Sleep Behavior Disorder. *Movement Disorders*. 2021;36(7):1720-1726. doi:10.1002/mds.28585

78. Waldthaler J, Stock L, Krüger-Zechlin C, Timmermann L. Age at Parkinson's disease onset modulates the effect of levodopa on response inhibition: Support for the dopamine

overdose hypothesis from the antisaccade task. *Neuropsychologia*. 2021;163:108082. doi:10.1016/j.neuropsychologia.2021.108082

79. Waldthaler J, Stock L, Student J, Sommerkorn J, Dowiasch S, Timmermann L. Antisaccades in Parkinson's Disease: A Meta-Analysis. *Neuropsychology Review*. 2021;31(4):628-642. doi:10.1007/s11065-021-09489-1

80. Hood AJ, Amador SC, Cain AE, et al. Levodopa slows prosaccades and improves antisaccades: An eye movement study in Parkinson's disease. *Journal of Neurology, Neurosurgery and Psychiatry*. 2007;78(6):565-570. doi:10.1136/jnnp.2006.099754

81. Waldthaler J, Tsitsi P, Svenningsson P. Vertical saccades and antisaccades: complementary markers for motor and cognitive impairment in Parkinson's disease. *npj Parkinson's Disease*. 2019;5(1):1-6. doi:10.1038/s41531-019-0083-7

82. Harsay HA, Buitenweg JIV, Wijnen JG, Guerreiro MJS, Ridderinkhof KR. Remedial effects of motivational incentive on declining cognitive control in healthy aging and Parkinson's disease. *Frontiers in Aging Neuroscience*. 2010;2(OCT):144. doi:10.3389/fnagi.2010.00144

83. MacAskill MR, Graham CF, Pitcher TL, et al. The influence of motor and cognitive impairment upon visually-guided saccades in Parkinson's disease. *Neuropsychologia*. 2012;50(14):3338-3347. doi:10.1016/j.neuropsychologia.2012.09.025

84. van Stockum S, MacAskill M, Anderson T, Dalrymple-Alford J. Don't look now or look away: Two sources of saccadic disinhibition in Parkinson's disease? *Neuropsychologia*. 2008;46(13):3108-3115. doi:10.1016/j.neuropsychologia.2008.07.002

85. Cameron IGM, Pari G, Alahyane N, et al. Impaired executive function signals in motor brain regions in Parkinson's disease. *Neuroimage*. 2012;60(2):1156-1170. doi:10.1016/j.neuroimage.2012.01.057

86. Dong W, Yan B, Johnson BP, et al. Ischaemic stroke: the ocular motor system as a sensitive marker for motor and cognitive recovery. *Journal of Neurology, Neurosurgery and Psychiatry*. 2013;84:337-341. doi:10.1136/jnnp-2012-304481

87. Filippopulos F, Eggert T, Straube A. Effects of cerebellar infarcts on cortical processing of saccades. *Journal of Neurology*. 2013;260(3):805-814. doi:10.1007/s00415-012-6708-y

88. Pierrot-Deseilligny C, Rivaud S, Gaymard B, Agid Y. Cortical control of reflexive visually-guided saccades. *Brain*. 1991;114(3):1473-1485. doi:10.1093/brain/114.3.1473

89. Pierrot-Deseilligny C, Müri RM, Ploner CJ, Gaymard B, Demeret S, Rivaud-Pechoux S. Decisional role of the dorsolateral prefrontal cortex in ocular motor behaviour. *Brain*. 2003;126(6):1460-1473. doi:10.1093/brain/awg148
